# Simultaneous STING and lymphotoxin-β receptor activation induces B cell responses in tertiary lymphoid structures to potentiate antitumor immunity

**DOI:** 10.1038/s41590-025-02259-8

**Published:** 2025-09-02

**Authors:** Junko Sawada, Yasuhiro Kikuchi, Maxwell Duah, Jose Luis Herrera, Fumiaki Kanamori, Krisztian Csomos, Tomoko Stansel, Nobuyoshi Hiraoka, Masayuki Yoshida, Jolan Walter, Carl F. Ware, Masanobu Komatsu

**Affiliations:** 1 Cancer and Blood Disorders Institute, Institute for Fundamental Biomedical Research, and Department of Surgery, Johns Hopkins All Children’s Hospital, and Department of Orthopaedic Surgery, Johns Hopkins University School of Medicine, St. Petersburg, FL USA; 2 Division of Pediatric Allergy/Immunology, University of South Florida at Johns Hopkins All Children’s Hospital, St. Petersburg, FL USA; 3 Division of Pathology and Clinical Laboratories, National Cancer Center Hospital; Division of Molecular Pathology, Analytical Pathology, National Cancer Center Research Institute, Tokyo, Japan; 4 Laboratory of Molecular Immunology, Infectious and Inflammatory Diseases Center, Sanford Burnham Prebys Medical Discovery Institute, La Jolla, CA USA; 5 Present Address: Translational Genetics and Genomics Section, National Institute of Arthritis and Musculoskeletal and Skin Diseases (NIAMS), National Institutes of Health (NIH), Bethesda, MD USA

**Keywords:** Tumour immunology, Cancer immunotherapy

## Abstract

B cell-rich tertiary lymphoid structures (TLS) are associated with favorable prognosis and positive response to immunotherapy in cancer. Here we show that simultaneous activation of innate immune effectors, STING and lymphotoxin-β receptor (LTβR), results in CD8^+^ T cell-dependent tumor suppression while inducing high endothelial venule development and germinal center-like B cell responses in tumors to generate functional TLS in a T cell-dependent manner. In a neoadjuvant setting, activation of STING and LTβR by their agonists effectively immunized mice against tumor recurrence, leading to long-term survival. STING activation alone was insufficient for inducing B cell-containing TLS or eliciting long-term therapeutic effects. However, when combined with LTβR activation, it improved the fitness of TLS with B cell expansion and maturation to IgG-producing long-lived plasma cells and memory cells, increased CD4^+^ T cell recruitment and memory CD8^+^ T cell expansion, and shifted the T_H_2/T_H_17 balance, resulting in the potentiation of humoral and cellular immunity against tumors. These findings suggest a therapeutic approach of simultaneously activating STING and lymphotoxin pathways.

## Abstract (web summary)

Sawada et al. show simultaneous activation of the STING and lymphotoxin beta receptor signaling induces B cell-activating germinal center responses within tumor environment and enhances antitumor responses.

## Main

The tumor microenvironment has a profound influence on the response to cancer therapy. TLS are often observed in lymphocyte-infiltrated ‘immune hot’ tumors. TLS are formed at the sites of chronic inflammation and resemble the secondary follicles of lymph nodes. These structures are composed of dense clusters of B cells containing follicular helper T (T_FH_) cells and follicular dendritic cells and surrounded by CD4^+^ and CD8^+^ T cells. TLS are typically found associated with high endothelial venules (HEVs), which are blood vessels normally found in the lymph nodes, to recruit naive B cells and T cells from the circulation^1–3^. Thus, TLS resemble germinal centers of inflamed lymph nodes, and they are believed to be active sites for lymphocyte stimulation by antigens for the selection, proliferation and maturation of effector B cells that produce high-affinity antibodies^4–6^. Acting locally within tumors, tumor-associated TLS may be central to the development of adaptive immunity against tumors, with direct access to tumor antigens^4–6^. TLS have been observed in various types of cancer, including melanoma, lung cancer, breast cancer and pancreatic cancer^5,7–10^, as well as rare cancer types such as rhabdomyosarcoma^11^. The high density of intratumoral TLS shows a positive correlation with patient survival^5,7–10^. The clinical evidence supports the importance of TLS for patients’ high response rate to immune checkpoint inhibition therapy^8–10^ and conventional chemotherapy^5–7^. These clinical studies indicated that the presence of abundant B cells associated with TLS is the most important prognostic factor in predicting patient survival, suggesting a vital role of intratumoral B cells in antitumor immunity^8–10^. Pharmacologic/biologic agents to promote the development of lymph node-like B cell germinal centers in TLS may provide an effective treatment for therapy-resistant cancers.

The normal development of lymphoid organs such as lymph nodes and Peyer’s patches requires LTβR signaling^12–16^. LTβR provides differentiation signals to lymphoid stromal cells, forming an effective architecture that promotes efficient immune responses^17–21^. LTβR signaling is essential for the development of HEVs, which serve as the gateway for lymphocytes to enter the lymphoid organs^22^. LTβR is also required for chemokine secretion, which recruits and organizes B lymphocytes and T lymphocytes and antigen-presenting dendritic cells into germinal centers. Activation of LTβR by its ligand LIGHT or agonistic antibodies has been shown to increase HEV formation and B lymphocyte and T lymphocyte infiltration into tumors^23–28^. However, the formation of mature TLS with germinal center responses was not demonstrated in previous studies. LTβR pathway may require cooperation with innate receptor signals to form mature TLS. The stimulator of interferon genes (STING) is an intracellular danger signal sensor responsible for the induction of type-I interferon (IFN)-stimulated genes, hence providing a crucial bridge between innate and adaptive immunity^29,30^. Spontaneous or chemotherapy-induced tumor cell death produces cyclic GAMP (cGAMP), an activating ligand for STING, which induces IFN and inflammatory cytokine responses in the tumor microenvironment^31,32^. A previous study showed that intratumoral administration of STING agonist ADU-S100 induced the formation of lymphoid aggregates composed of CD3^+^ T cells and dendritic cells in subcutaneous mouse melanoma^33^. However, these lymphoid structures lacked germinal center-like B cell clusters characteristic to TLS, demonstrating that STING activation alone is insufficient for promoting the development of functional TLS^33^. To date, there have been no reports of therapeutic induction of TLS in patients’ tumors in clinical trials of STING agonists or other cancer therapeutics. The impact of innate immune activation and engagement of the adaptive immune system in the cascade of mechanisms required for the intratumoral TLS formation has yet to be fully explored. Our approach here focuses on defining the relationship of LTβR and STING in inducing functional TLS and antitumor immune responses.

## Results

### Microenvironmental cues associated with TLS

**Extended Data Fig. 1:**
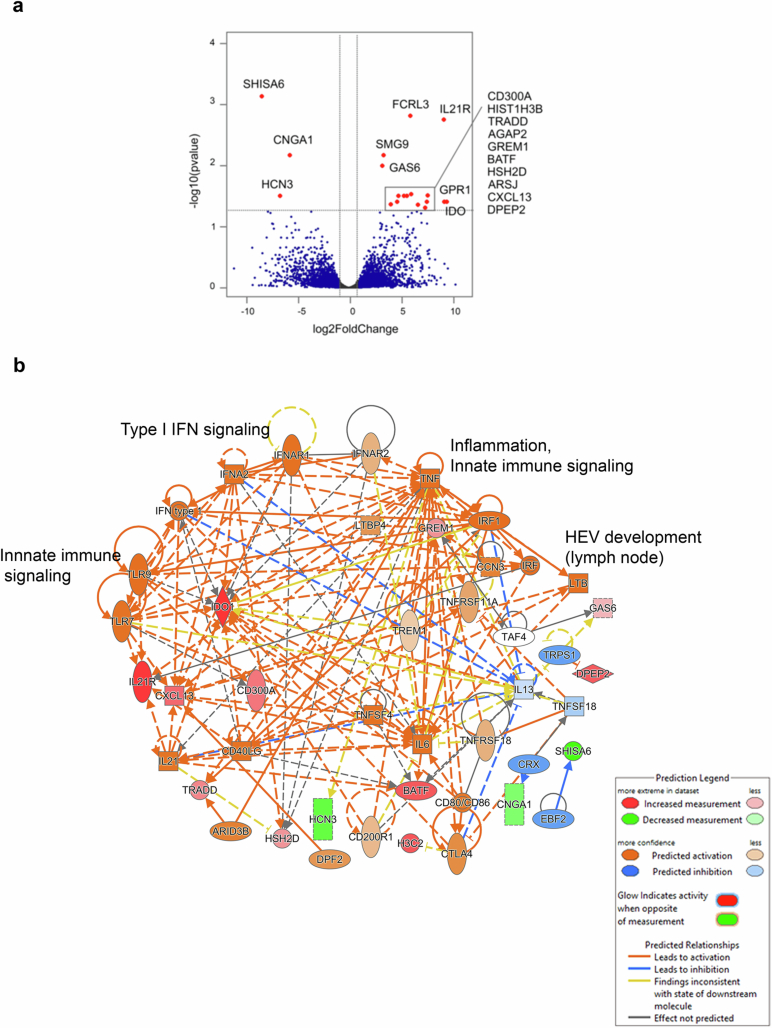
Pathway analysis based on the differential transcriptome of tumor vascular endothelium in TLS-rich tumors. A previous transcriptome analysis of human breast adenocarcinoma vasculature identified differentially expressed genes between non-HEV endothelium of TLS-rich vs. TLS-free tumors^34^. **a**, Volcano plot presentation of differentially expressed genes. The log2 fold change of gene expression in the endothelium of TLS-rich tumors compared with TLS-free tumors is shown on the X-axis. P values are shown on the Y-axis (-log10). The genes with statistically significant differences are shown in red dots. p < 0.05. **b**, An upstream prediction analysis of these gene sets was performed using the Ingenuity Pathway Analysis (IPA) program. This study identified Type-I IFN, tumor necrosis factor (TNF), Toll-like receptor 7 (TLR7), TLR9, and lymphotoxin-β (LTβ) suggesting inflammation and innate immunity signaling in the tumor microenvironment. Also identified were CXCL13, CD40L, IL21, and IL21R, which regulate B cell recruitment, activation, and differentiation as well as generation/maintenance of follicular helper T (TFH) cells and germinal center development.

Previously, we conducted a transcriptome analysis of tumor vasculature in human breast cancer, which identified several genes differentially expressed between the endothelium of tumors rich in TLS and those tumors lacking TLS^34^. The differences in gene expression patterns are likely caused by the exposure of endothelial cells to different environmental cues in TLS-rich tumors versus TLS-free tumors. We reasoned the differentially regulated signaling pathways in endothelial cells may identify the nature of these environmental cues. Based on this idea, we performed an upstream prediction analysis of these gene sets using the ingenuity pathway analysis program. Our assessment identified transcripts of type-I IFN and other signaling molecules involved in inflammation and innate immunity, including tumor necrosis factor (TNF), Toll-like receptor 7 (TLR7), TLR9 and lymphotoxin-β (LTβ; Extended Data Fig. 1). Also identified were regulators of B cell recruitment, activation and differentiation as well as generation/maintenance of T_FH_ cells and germinal center development, such as CXCL13, CD40L, interleukin (IL)-21 and IL-21R, consistent with the development of TLS (Extended Data Fig. 1). The involvement of LTβ and genes responding to LTβR signaling, such as *CXCL13*, suggest an active role of the LTβ–LTβR pathway in tumor-associated endothelium and lymphoid organogenesis as previously reported^12–16,23–28^. The involvement of type-I IFN suggests both innate and adaptive immune pathways are active in the tumor microenvironment.

### Drug induction of tumor-associated TLS

**Fig. 1:**
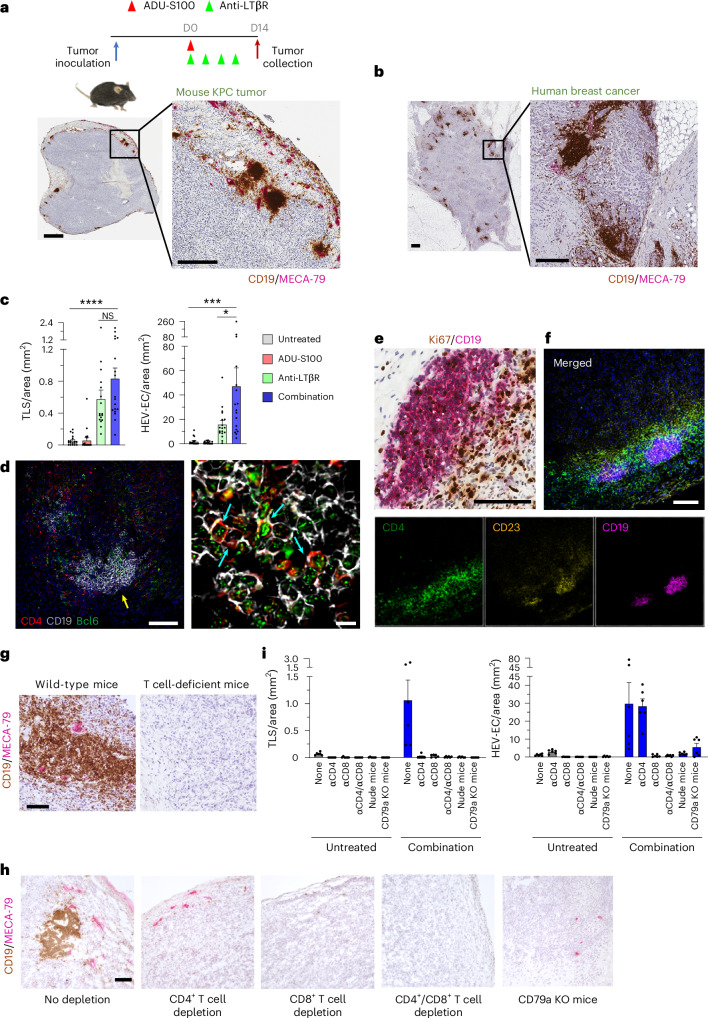
Drug induction of TLS and HEVs in subcutaneous tumors. **a**, STING agonist ADU-S100 (2 μg intratumoral) and/or anti-LTβR agonistic antibody (100 μg, intraperitoneal (i.p.)) were administered to mice bearing subcutaneous KPC tumors as monotherapy or in combination as indicated. Immunohistochemistry of B cells (CD19, brown) and HEVs (MECA-79, magenta) shows numerous TLS induced by combination therapy (day 14). Scale bars, 1 mm (low magnification) and 100 μm (high magnification). **b**, CD19 and MECA-79 staining of TLS-rich human breast adenocarcinoma for comparison. Scale bars, 1 mm (low magnification) and 100 μm (high magnification). **c**, KPC tumor sections of different treatment groups were stained for CD19 and MECA-79, scanned for the whole tumor area, and the number of TLS and HEV endothelial cells (HEV-ECs) were quantified per tumor area (mm^2^) by Halo image analyses. The results of two independent experiments were combined to generate the graphs. A total of *N* = 17–19 tumors were analyzed. *P < 0.05, ***P < 0.001, ****P < 0.0001. **d**, Left: immunofluorescence confocal image (*z*-stack) of TLS induced by combination therapy. Scale bar, 100 μm. Right: the TLS area indicated by the arrow in the left image is shown in higher magnification (three-dimensional confocal image). Scale bar, 10 μm. Nuclear staining of germinal center/follicular cell transcription factor Bcl6 (green) is surrounded by B cell surface marker CD19 (white) or helper T cell marker CD4 (red), indicating germinal center B cells or T_FH_ cells, respectively. Arrows indicate T_FH_ cells found among the dense B cell cluster, demonstrating the intimate interaction between the T_FH_ cell surface (red) and the B cell surface (white). **e**, Proliferating B cells were detected in TLS by Ki-67 and CD19 double staining. Scale bar, 100 μm. **f**, Triple immunofluorescence staining of LTβR monotherapy-induced TLS for CD4, CD19 and CD23 (a follicular cell marker) shown in merged and individual color channels. Blue, DAPI. Scale bar, 100 μm. **g**, Combination therapy on KPC tumors grown in wild-type or T cell-deficient nude mice (C57BL/6 background) demonstrating that neither TLS nor HEVs formed in tumors in the absence of T cells. A representative image of 16 tumors is shown for each genotype. Scale bar, 100 μm. **h**, CD4^+^ T cells and/or CD8^+^ T cells are depleted in wild-type mice by i.p. injection of depleting antibodies one or two days before KPC tumor inoculation as described in Extended Data Fig. 3a. Isotype IgG was injected for the no-depletion control group. Alternatively, KPC tumors were grown in B cell-deficient CD79a knockout mice and treated as shown in **a**. The development of TLS and HEVs was studied in these mice after combination therapy. A representative image of six tumors is shown for each group. Scale bar, 100 μm. **i**, The number of TLS and HEV-ECs were quantified per tumor area (mm^2^). *N* = 6. *P* < 0.0001. Representatives of at least three independent experiments are shown in **d**–**f**. Data are presented as the mean ± s.e.m. and analyzed using one-way analysis of variance (ANOVA) with Tukey’s test for statistical significance. All replicates represent biological replicates. NS, not significant.

**Extended Data Fig. 2:**
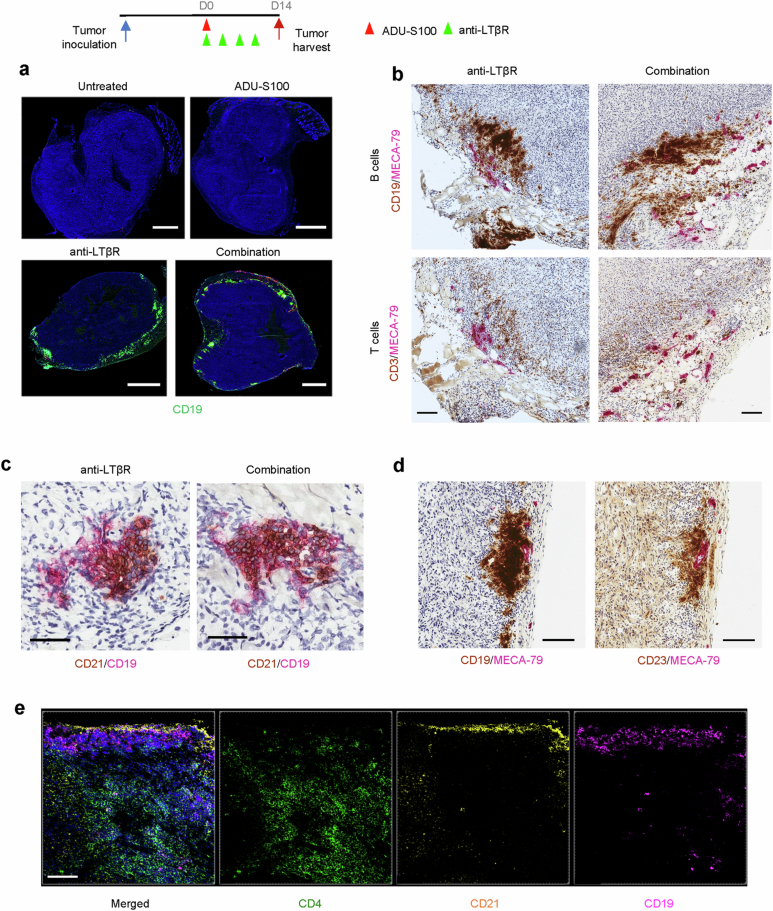
Formation of tumor-associated TLS upon agonist treatment. **a**, Cryosections of subcutaneous KPC tumors of different treatment groups were stained for CD19. The IHC images were deconvoluted to highlight the positive staining in bright green in low magnification to show whole tumor areas. Scale bar, 1 mm. **b**, Immunostaining of CD19 and CD3 with MECA-79 to visualize B cells, T cells, and HEVs in TLS areas of KPC tumors treated with LTβR agonist or LTβR/STING agonist combination. **c**, KPC tumors treated with anti-LTβR monotherapy or combination therapy. Areas of tumor sections containing TLS were stained for a germinal center marker CD21 (brown) and B cell marker CD19 (magenta). **d**, Left: TLS was stained for CD19 for B cells (brown) and MECA-79 for HEVs (magenta) in LTβR monotherapy-treated KPC tumors. Right: A consecutive tumor section was stained for a germinal center cell marker CD23 (brown). **e**, Triple immunofluorescence staining of TLS for CD4, CD21, and CD19 in LTβR monotherapy-treated KPC tumors. Blue, Hoechst. Scale bar 100 μm.

**Extended Data Fig. 3:**
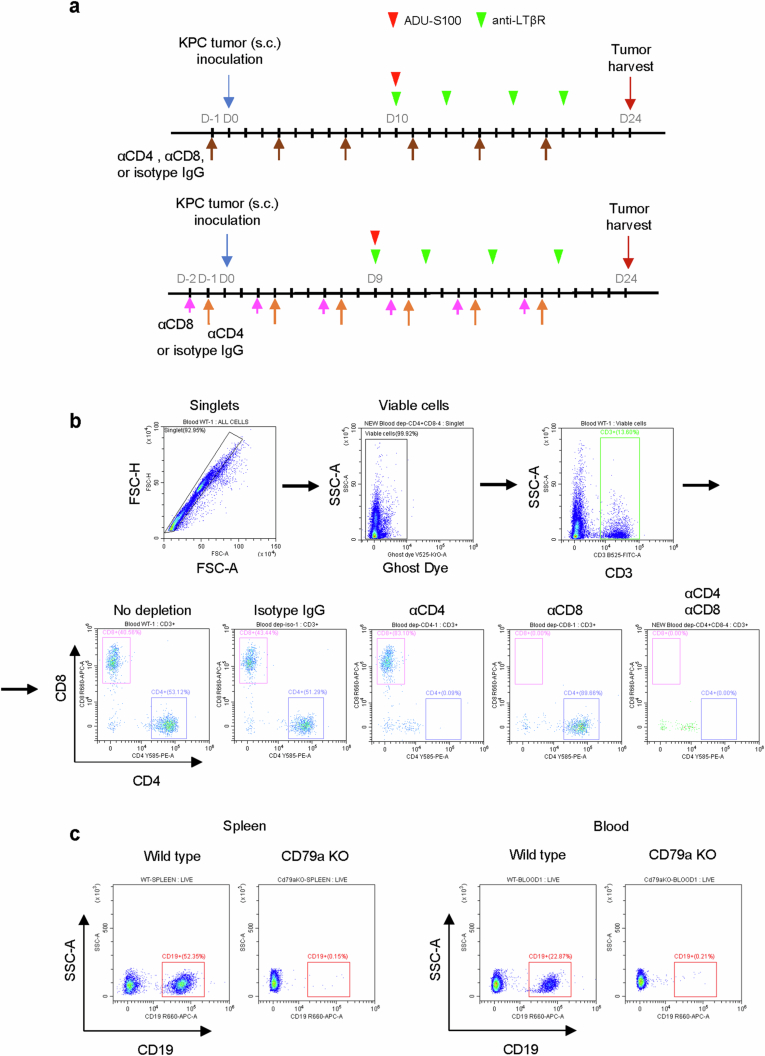
Agonist treatment study in T cell-depleted mice and B cell-deficient mice. **a**, Treatment schedule for subcutaneous KPC tumors. CD4^+^ and/or CD8^+^ T cells were deleted individually or together by i.p. injection of anti-CD4 and/or CD8 IgG (200 μg) every 4 days, starting from one or two days before tumor inoculation as indicated. Isotype IgG2b was injected for the no-depletion control group. **b**, T cell depletion was confirmed by FACS analysis of peripheral blood lymphocytes. Rat anti-mouse CD4 (clone: GK1.5) and rat anti-mouse CD8α (clone: 2.43) were used to deplete CD4^+^ and CD8^+^ T cells, respectively. Rat IgG2b was used as an isotype control (clone: LTF-2). **c**, Flow cytometry analyses of spleen and peripheral blood leukocytes show the absence of CD19^+^ B cells in CD79a knockout mice.

Based on these findings, we administered STING agonist ADU-S100 (cGAMP analog) and LTβR agonistic antibody (4H8) to tumor-bearing mice to examine whether activation of STING and LTβR pathways will induce TLS formation in TLS-free tumors by reproducing the microenvironment of TLS-rich tumors. For this study, we treated C57BL/6 mice bearing subcutaneous syngeneic tumors derived from *Kras*^*LSL.G12D/+*^*Trp53*^*LSL.R172H/+*^*Pdx1*-Cre mice (hereafter KPC tumors)^35^. These mice were treated with STING agonist alone, LTβR agonist alone or the two agonists in combination when tumors reached approximately 100 mm^3^ in volume. The STING agonist was administered once via intratumoral injection at 2 μg per tumor (day 0), and 100 μg anti-LTβR was administered intraperitoneally every 3–4 days, for a total of four times until day 10. Untreated mice or mice treated with the STING agonist rarely generated lymphoid aggregates in their tumors (Extended Data Fig. 2a). In comparison, mice treated with the LTβR agonist monoclonal antibody or in combination with the STING agonist induced numerous TLS that resembled human cancer TLS composed of dense clusters of B cells surrounded by CD3^+^ T cells and HEV vessels (Fig. 1a–c and Extended Data Fig. 2a,b). All mice in these two treatment groups developed TLS. The majority of B cells in these tumors were found in dense clusters as TLS unlike T cells, which were found in high density around TLS but also observed broadly throughout the tumor area. Many of these B cells expressed the germinal center B cell marker Bcl6 (Fig. 1d) and were in a proliferative state as indicated by Ki-67 staining (Fig. 1e). These lymphoid structures were also positive for follicular markers CD21 and CD23 (Fig. 1f and Extended Data Fig. 2c–e) and contained Bcl6^+^CD4^+^ T_FH_ cells (Fig. 1d), which are all characteristics of mature TLS in human cancers, distinct from immature lymphocyte aggregates^4,8^. Notably, neither TLS nor HEVs developed in T cell-deficient nude mice that were treated with agonist combination therapy, and B cell infiltration was extremely rare (Fig. 1g). We depleted CD4^+^ or CD8^+^ T cells individually or together in wild-type animals to further investigate the role of T cells (Extended Data Fig. 3a,b). This study showed that both CD4^+^ T cells and CD8^+^ T cells are essential to TLS formation. The depletion of either CD4^+^ T cells or CD8^+^ T cells nearly abrogated TLS formation (Fig. 1h,i). Interestingly, CD4^+^ T cell depletion did not affect HEV formation while CD8^+^ T cell depletion abrogated HEVs, indicating that the HEV formation alone does not promote TLS development in the absence of CD4^+^ T cells. Depletion of both T cell subsets completely abrogated TLS and HEV formations confirming the result of the nude mouse study (Fig. 1g–i). These observations demonstrate the essential role of T cells in creating the immune landscape supportive of antitumor B cell responses in the form of TLS. TLS did not develop upon combination therapy in B cell-deficient CD79a knockout mice as expected (Fig. 1h,i and Extended Data Fig. 3c). Interestingly, HEVs developed in these mice but at a considerably reduced density, indicating that B cells are also crucial for HEV formation (Fig. 1h,i).

**Fig. 2:**
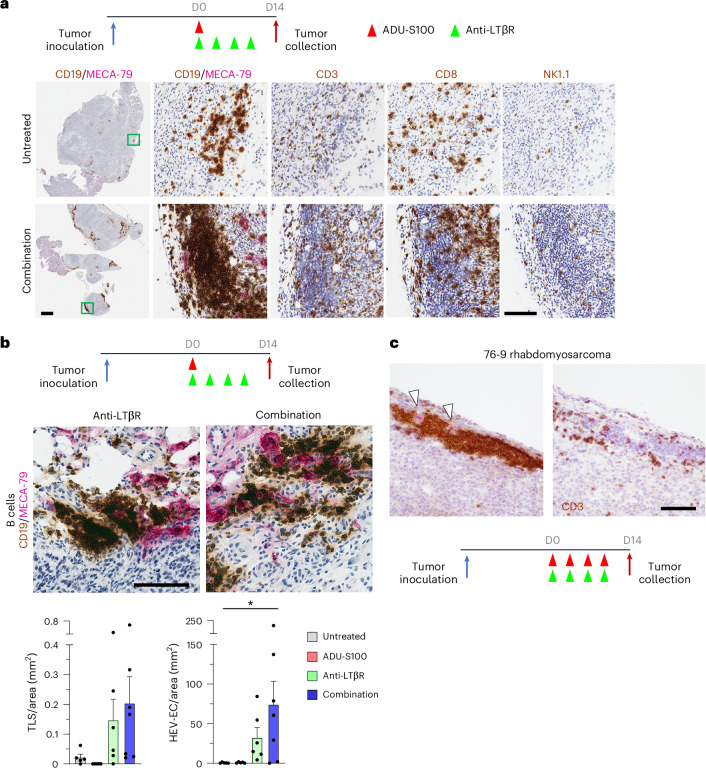
Drug induction of TLS and HEVs in orthotopic tumors. **a**, TLS formation in orthotopic KPC tumors in the pancreas. B cells (CD19), T cells (CD3), cytotoxic T cells (CD8) and NK cells (NK1.1) were stained along with HEVs (MECA-79) in serial sections of orthotopic pancreatic tumors 14 days after the start of combination therapy. The areas indicated by the squares in the first images to the far left are shown in high magnification in the images to the right. Representative images of 3–4 tumors are shown. Scale bars, 1 mm (left) and 100 μm (high-magnification images). **b**, TLS (brown) and HEVs (magenta) formed in orthotopic Py230 mouse mammary tumors upon agonist treatment. Representative images of 6–7 tumors are shown. Graphs show the TLS and HEV-EC densities in tumors of different treatment groups. Stained sections were scanned for the whole tumor area, and the number of TLS and HEVs were quantified per tumor area (mm^2^) by Halo image analyses. *N* = 5–7 tumors from 2 independent experiments representing biological replicates. Data are presented as the mean ± s.e.m. and analyzed using one-way ANOVA with Tukey’s test for statistical significance. *P < 0.05. Scale bar, 100 μm. **c**, Left: TLS formation (brown, CD19) in orthotopic 76-9 rhabdomyosarcoma in the calf muscle. Arrowheads, HEVs (magenta, MECA-79). Right: CD3 staining of a consecutive section. Combination therapy was given four times as indicated. Representative images of ten treated tumors are shown. Scale bar, 100 μm.

In wild-type mice, TLS and HEV formation was also observed in orthotopic KPC tumors grown in the pancreas (Fig. 2a) and in orthotopic Py230 (MMTV-PyMT) mammary tumors (Fig. 2b) as well as in ‘immune cold’ orthotopic 76-9 rhabdomyosarcoma grown in the calf muscle upon combination therapy (Fig. 2c). Either LTβR monotherapy or combination therapy induced TLS in KPC and Py230 tumors (Figs. 1c and 2b). In comparison, combination therapy with repeated administration of STING agonist was necessary for TLS development in rhabdomyosarcomas, suggesting the importance of STING activation for TLS induction in ‘immune cold’ tumors (Fig. 2c).

**Extended Data Fig. 4:**
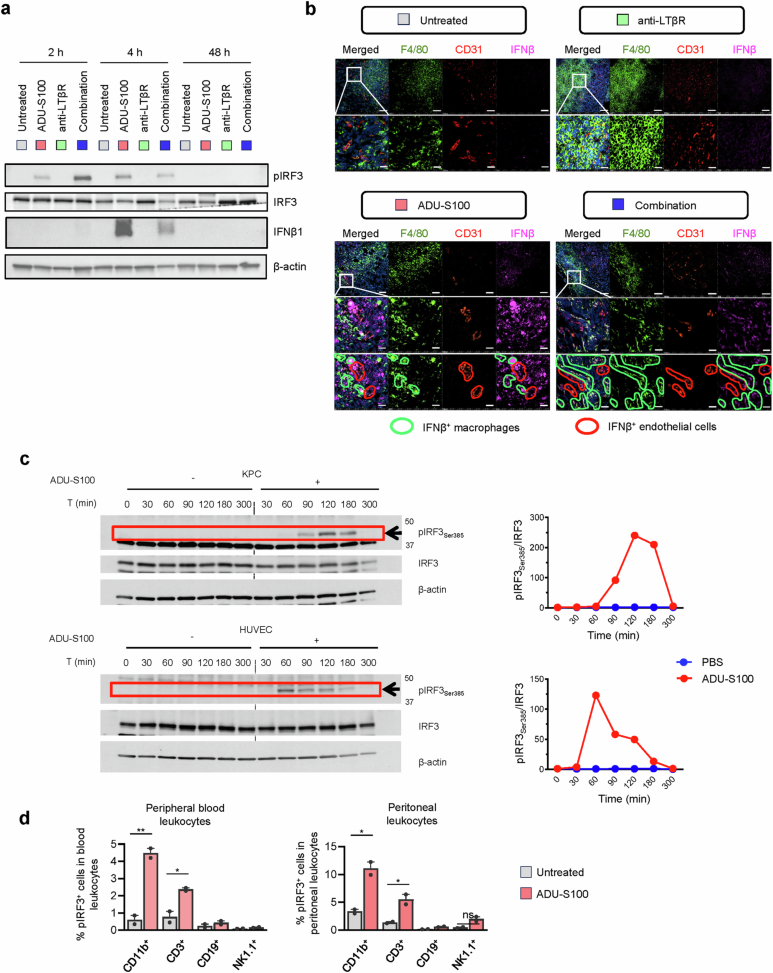
Induction of STING signaling by intratumoral ADU-S100. **a**, KPC tumor-bearing mice were treated with/without agonist monotherapy or combination therapy, and tumors were harvested at indicated timepoints. Two tumors were pooled and lysed for western blot analysis for each treatment option at each timepoint. The activation of STING pathway was analyzed by phosphorylation of IRF3 and IFNβ expression downstream of STING. A representative western blot of two independent experiments is shown. β-actin was used as a loading control. **b**, KPC tumors were co-stained for IFNβ with macrophage (F4/80) and endothelial cell (CD31) markers at 4 h post monotherapy or combination therapy to evaluate STING activation in these cells. Immunofluorescence images (top panels) are shown in higher magnification in the middle and/or bottom panels. IFNβ^+^ macrophages and endothelial cells are indicated by green and red circles, respectively. Scale bar, 100 μm (top panels) and 20 μm (middle and bottom panels). **c**, ADU-S100 (10 μM) was added to the culture of KPC cells or endothelial cells (HUVEC) for indicated time, and the activation of STING signaling was analyzed by IRF3 Ser385 phosphorylation. The quantification of phosphorylated IRF3 was normalized by total IRF3. β-actin was used as a loading control. **d**, Leukocytes were isolated from peripheral blood or the peritoneum of normal mice and incubated *in vitro* with 7.2 μg/ml STING agonist ADU-S100 for 2 h at 37 °C. Phosphorylation of IRF3 in leukocyte subsets (CD11b^+^ myeloid cells, CD3^+^ cells, CD19^+^ cells, and NK1.1^+^ cells) was detected by α-phospho-IRF3 Ser385 antibody staining. Cells were permeabilized using eBioscience™ Transcription Factor Staining Buffer Set before staining. The percent fraction of phospho-IRF3^+^ cells in each subset of blood or peritoneal leukocytes was determined by flow cytometry. N = 2 *p < 0.05, **p < 0.01. Source data

We next analyzed how tumors respond to the STING agonist treatment. KPC tumors were collected at different time points after agonist treatment, and STING signaling was analyzed by western blot. Intratumoral ADU-S100 induced IRF3 phosphorylation and IFNβ expression within 4 h, indicating the rapid activation of the STING pathway in the tumor microenvironment (Extended Data Fig. 4a). Immunofluorescence of tumor sections confirmed IFNβ induction in macrophages, endothelial cells and possibly other cell types 4 h after ADU-S100 monotherapy or combination therapy (Extended Data Fig. 4b). We also analyzed STING signaling activation by ADU-S100 in different cell types in vitro. The phosphorylation of IRF3 in cultured KPC cells and endothelial cells was analyzed by western blot (Extended Data Fig. 4c). Leukocytes from peripheral blood or peritoneum of normal mice were analyzed by flow cytometry (Extended Data Fig. 4d). The results of these studies suggest that multiple cell types, including tumor cells, endothelial cells, T cells and macrophages/monocytes, could respond to intratumoral ADU-S100 and collectively contribute to the STING-induced immune responses.

### Neoadjuvant combination therapy inhibits tumor growth and recurrence

**Fig. 3:**
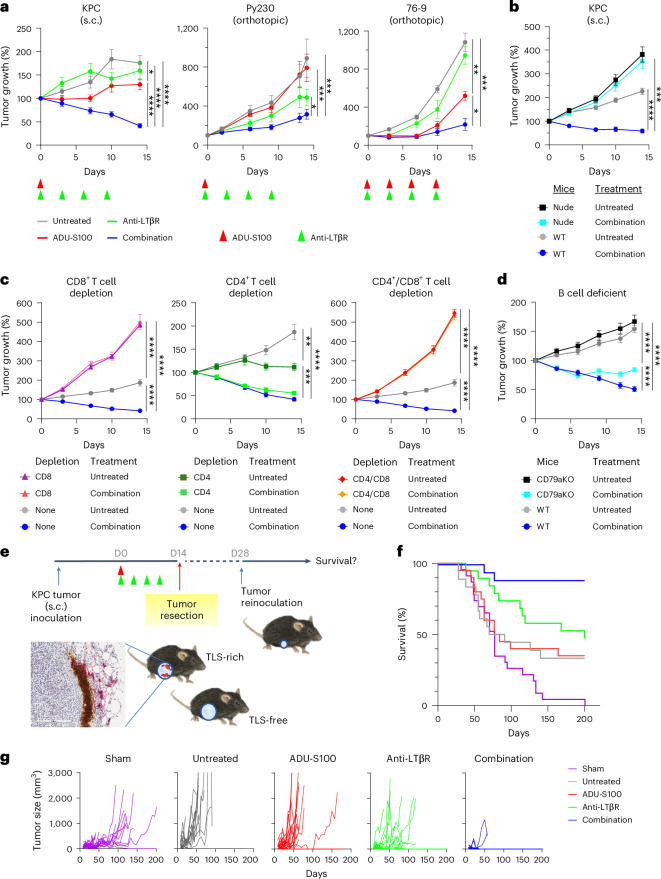
Therapeutic effects of neoadjuvant agonist combination on tumor growth and survival. **a**, Mice bearing different tumor types were treated with STING agonist ADU-S100 (2 μg intratumoral) and/or anti-LTβR agonistic antibody (100 μg, i.p.) as indicated (arrowheads), and the tumor growth curves were monitored until surgical resection. s.c., subcutaneous. *N* = 22–28 (KPC), *N* = 11–18 (Py230), *N* = 5 (76-9) tumors from 2 independent experiments combined, representing biological replicates. *P < 0.05, **P < 0.01, ***P < 0.001, ****P < 0.0001. **b**, Subcutaneous KPC tumor growth in wild-type (WT) or T cell-deficient nude mice treated or untreated with combination therapy. *N* = 10 or 12. **c**, CD4^+^ T cells and/or CD8^+^ T cells are depleted in combination therapy-treated and untreated mice by i.p. injection of depletion antibodies one or two days before KPC tumor inoculation (s.c.) as described in Extended Data Fig. 3a. Isotype IgG was injected to the no-depletion control group. Tumor growth was monitored for 14 days. *N* = 10. **d**, Subcutaneous KPC tumor growth in WT or B cell-deficient CD79a knockout mice treated or untreated with combination therapy. *N* = 20. **e**, Treatment schedule of neoadjuvant agonist therapies for s.c. KPC tumors and tumor resection followed by tumor reinoculation. The primary tumors were either TLS-rich or TLS-free at the time of the resection, depending on the neoadjuvant treatment options. **f**, Survival curves after tumor reinoculation. Sham control mice had no primary tumors but received tumor inoculation for the first time 2 weeks after sham surgery. *N* = 18–23. **g**, Growth curves of reinoculated tumors. For **a**–**g**, the results of two or more independent experiments were combined and presented in the graphs. Data in **a**–**d** are presented as the mean ± s.e.m. and analyzed using two-way ANOVA with Tukey’s test for statistical significance. All replicates represent biological replicates. Source data

The growth of different types of tumors was monitored for 2 weeks from the day of treatment (day 0). The LTβR monotherapy had little or no significant effect, and only a moderate effect of STING monotherapy was observed in the KPC tumors and Py230 mammary tumors at this dose (2 μg intratumoral; Fig. 3a). In comparison, the combination therapy reduced KPC tumor burden by more than 50% and significantly delayed Py230 and 76-9 tumor growth (Fig. 3a). The combination therapy showed no therapeutic benefit in T cell-deficient nude mice (Fig. 3b) suggesting that T cells play an essential role in the immediate-early tumor inhibition by this therapy. The lack of effect in nude mice also indicates that the agonist treatment has little or no direct inhibitory effect on tumor growth. The depletion of CD8^+^ T cells or simultaneous depletion of CD4^+^ T and CD8^+^ T cells resulted in exaggerated tumor growth in both combination therapy-treated and untreated mice, and combination therapy had no tumor suppression effect, demonstrating the importance of CD8^+^ T cells (Fig. 3c). On the other hand, the depletion of CD4^+^ T cells inhibited tumor growth in untreated mice after day 7 (Fig. 3c). This inhibition is likely due to the depletion of CD4^+^ regulatory T cells and resulting potentiation of CD8^+^ T cells^36^, as all CD4^+^ T cells were depleted in this procedure (Extended Data Fig. 3b). In comparison with these mice, the CD4^+^ T cell-depleted, combination therapy-treated mice did not show additional tumor suppression from day 7 (Fig. 3c), which may be related to the inability of CD4^+^ T cell-depleted mice to develop TLS. Therefore, we tested the combination therapy in B cell-deficient CD79a knockout mice to assess the contribution of TLS-B cells. In these mice, combination therapy was unable to continue to reduce tumor burden after day 6, indicating a partial loss of the therapeutic effect (Fig. 3d). These results demonstrated that the initial tumor inhibition is mainly mediated by CD8^+^ T cells, with a delayed and moderate contribution of B cells starting in the second week.

The role of B cells in TLS is thought to be the local development of humoral immunity involving antibody productions and differentiation of memory B cells; hence, contributions of TLS-B cells cannot be fully examined in a short-term, 2-week tumor study. To investigate the importance of TLS and humoral immunity, we used a tumor recurrence model. In this model, we surgically resected the subcutaneous KPC tumors and sentinel lymph nodes after monotherapy or combination therapy on day 14 and reinoculated KPC cells subcutaneously 2–3 weeks later to mimic tumor relapses (Fig. 3e). Remarkably, mice that received the neoadjuvant agonist combination before the tumor resection, which all developed TLS, showed excellent long-term survival with complete disappearance of the tumors (Fig. 3f,g). The reinoculated tumor cells initially grew in the host mice as small papules at the injection sites for several days but then regressed and eventually disappeared, except for a few cases, demonstrating that these animals became resistant to KPC tumors. Mice that received neoadjuvant LTβR monotherapy, which also developed TLS, acquired tumor resistance but of lesser duration (Fig. 3f,g). The neoadjuvant STING monotherapy had little survival benefit, and the reinoculated tumors grew rapidly in most mice, similarly to the untreated control.

### Accumulation of activated B cells in tumors

**Fig. 4:**
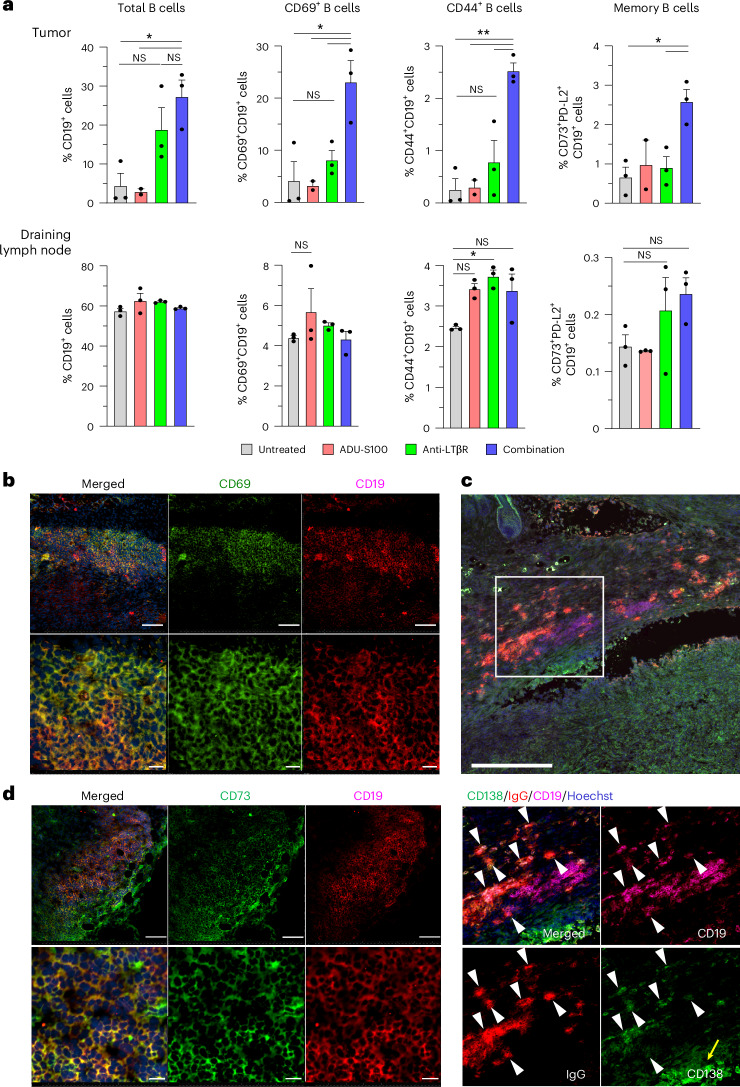
Drug-induced TLS harbor antigen-primed class-switched plasma and memory B cells. **a**, Upper bar charts: the abundance of B cells in primary KPC tumors on the day of tumor resection (day 14) determined by flow cytometry. Analyses of total B cells (CD19) as well as CD69 and CD44 expression were conducted to determine early and late activation states of B cells, respectively. The abundance of total and activated B cells is presented as a percentage fraction of the total gated lymphocytes. The fraction of memory B cells was determined by CD73/PD-L2/CD19 triple staining. For each data point, three tumors were pooled and dissociated to isolate lymphocytes for the FACS analysis. A total of 6–9 tumors were examined. Data are presented as the mean ± s.e.m. and analyzed using one-way ANOVA with Tukey’s test for statistical significance. *P < 0.05; **P < 0.01. Lower bar charts: similar analyses of tumor-draining lymph nodes on day 14. For each data point, six draining (inguinal) lymph nodes were pooled for FACS analysis. A total of 18 lymph nodes were examined for each group. A representative of two independent experiments is shown for each analysis. **b**, CD69 and CD19 immunofluorescence of combination therapy-treated KPC tumors showing that the majority of B cells in TLS are activated. Scale bars, 100 μm (upper images) and 20 μm (lower images). **c**, Accumulation of IgG-expressing CD138^+^ plasma cells in TLS (arrowheads). CD138 (syndecan-1) is also expressed by tumor cells (yellow arrow). The TLS area indicated by the square is shown in higher magnification in the lower images. Scale bar, 300 μm. **d**, CD73^+^CD19^+^ B cells are present in TLS. The area of CD73/CD19 doubly stained cells is shown in higher magnification in the lower images. Scale bars, 100 μm (upper images) and 20 μm (lower images). Representatives of at least three independent experiments are shown in **b**–**d**.

**Fig. 5:**
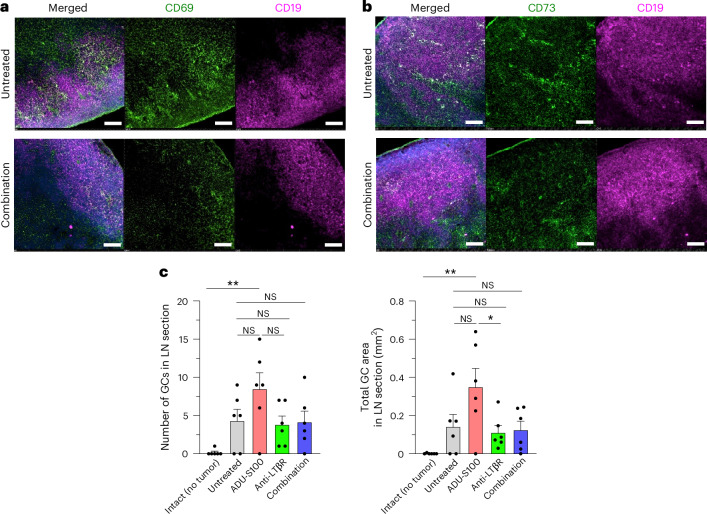
Germinal centers of tumor-draining lymph nodes. Tumor-draining lymph nodes were collected on day 14 and frozen sections examined by immunofluorescence of lymphoid follicles. **a**, Staining of lymph node follicles of combination therapy-treated and untreated control mouse groups with CD19 (magenta) and CD69 (green) antibodies to assess the activation of follicular B cells in these lymph nodes. Representative sections of six lymph nodes are shown for each group. Scale bars, 100 μm. **b**, Similarly, staining with CD19 (magenta) and CD73 (green) antibodies to detect memory B cells in the lymph node follicles of combination therapy-treated and untreated control mice. Scale bars, 100 μm. **c**, Clusters of Ki-67^+^ B cells within CD19^+^ B cell follicles were recognized as germinal centers (GCs) by immunostaining of each lymph node section. The number of GCs (left) and the total area of GCs (right) in each lymph node (LN) section are presented in the graphs. Inguinal lymph nodes of mice without tumor implantation were used for intact lymph node control. *N* = 6, *P < 0.05, **P < 0.01.

**Extended Data Fig. 5:**
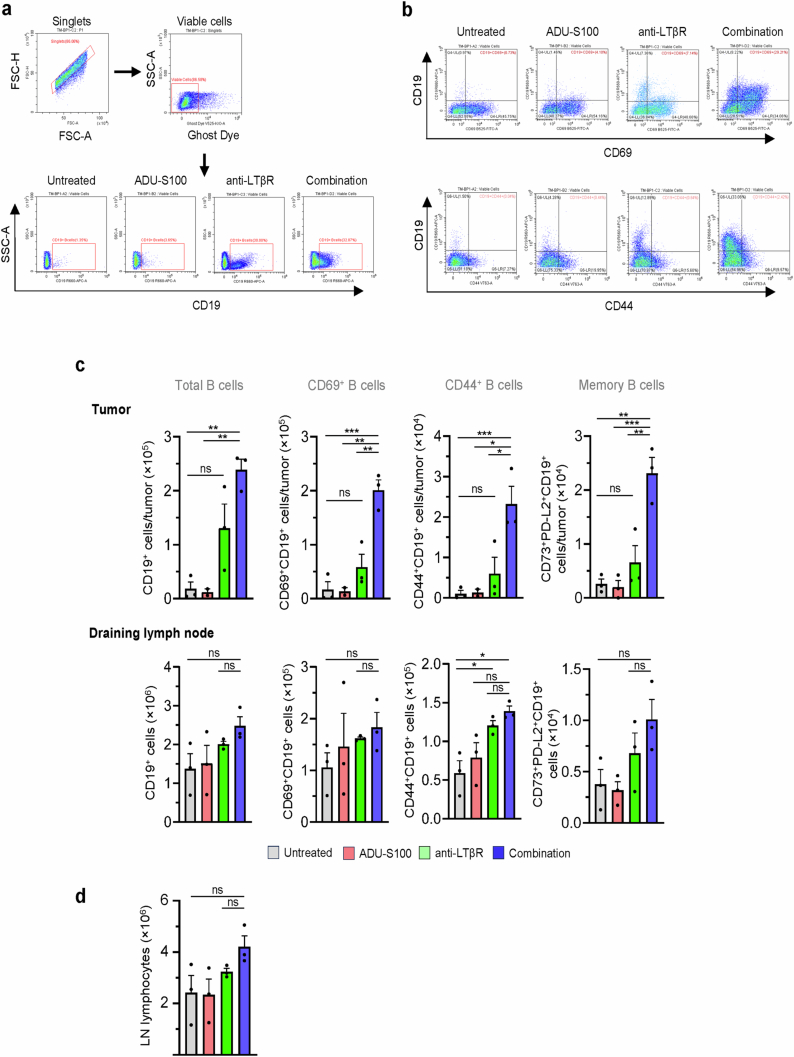
Flow cytometry analyses of tumor-infiltrating B cells. **a**, KPC tumors were resected on Day 14 of the treatment study, dissociated into single cells in suspension by Liberase digestion, and stained for CD19, CD69, and CD44. Viable lymphocytes were then analyzed by FACS. **b**, Expression of early activation marker CD69 and late activation marker CD44 in tumor-infiltrating B cells. **c**, B cells count in tumor and draining lymph node. Upper panels: The number of total CD19^+^ B cells or activated B cells per tumor was calculated from the total live cell count after tumor dissociation and the % fractions of these B cells as determined by flow cytometry (Fig. 4a). For each data point, three tumors were pooled, and the obtained cell number was divided by three to yield average B cell count/tumor. Total 6-9 tumors were examined. Lower panels: Similarly, the number of B cells in the draining lymph node was calculated from the total lymphocyte count in each lymph node and the % fraction of B cells in them. A representative result of two independent experiments is shown for each analysis. *p < 0.05; ***p < 0.01; **p < 0.001; ns, not significant. **d**, Total lymphocyte count in a draining lymph node. For each data point, six draining lymph nodes were pooled and averaged.

To explore the mechanism by which this neoadjuvant therapy confers resistance to future tumor development, we further examined tumor-infiltrating lymphocytes and TLS in the primary tumors on the day of tumor resection. Our flow cytometry analysis showed that tumors of the combination therapy group had a significant increase in B cells, consistent with the presence of TLS in these tumors (Fig. 4a and Extended Data Fig. 5a–c). B cells were prominent (average 27% of infiltrating lymphocytes) in tumors treated with the STING–LTβR combination therapy (Fig. 4a). Mice treated with LTβR monotherapy also showed an increase in B cells. The analyses of early and late activation markers, CD69 and CD44, indicated that activated B cells were substantially increased in tumors after combination therapy but not LTβR monotherapy (Fig. 4a and Extended Data Fig. 5c). In addition, a significant increase in CD73^+^PD-L2^+^CD19^+^ memory B cells was observed in tumors after combination therapy but not LTβR monotherapy (Fig. 4a and Extended Data Fig. 5c). Immunofluorescence of tumor sections confirmed that most B cells in TLS were activated in the combination therapy group (Fig. 4b). In contrast, the fractions of total and activated B cells were not significantly different between the tumor-draining lymph nodes of three therapy groups (Fig. 4a). There was a trend toward an increase in the B cell counts in lymph nodes upon LTβR monotherapy or combination therapy, reflecting a trend toward an increase in total lymphocyte counts in these lymph nodes (Extended Data Fig. 5d). This observation suggests that agonist treatments also influence lymph node responses to tumors. However, CD69, CD73 or Ki-67 staining of CD19^+^ B cell follicles did not indicate increased germinal center formation or activity in the lymph nodes upon combination therapy compared with other groups of tumor-bearing mice (Fig. 5).

**Extended Data Fig. 6:**
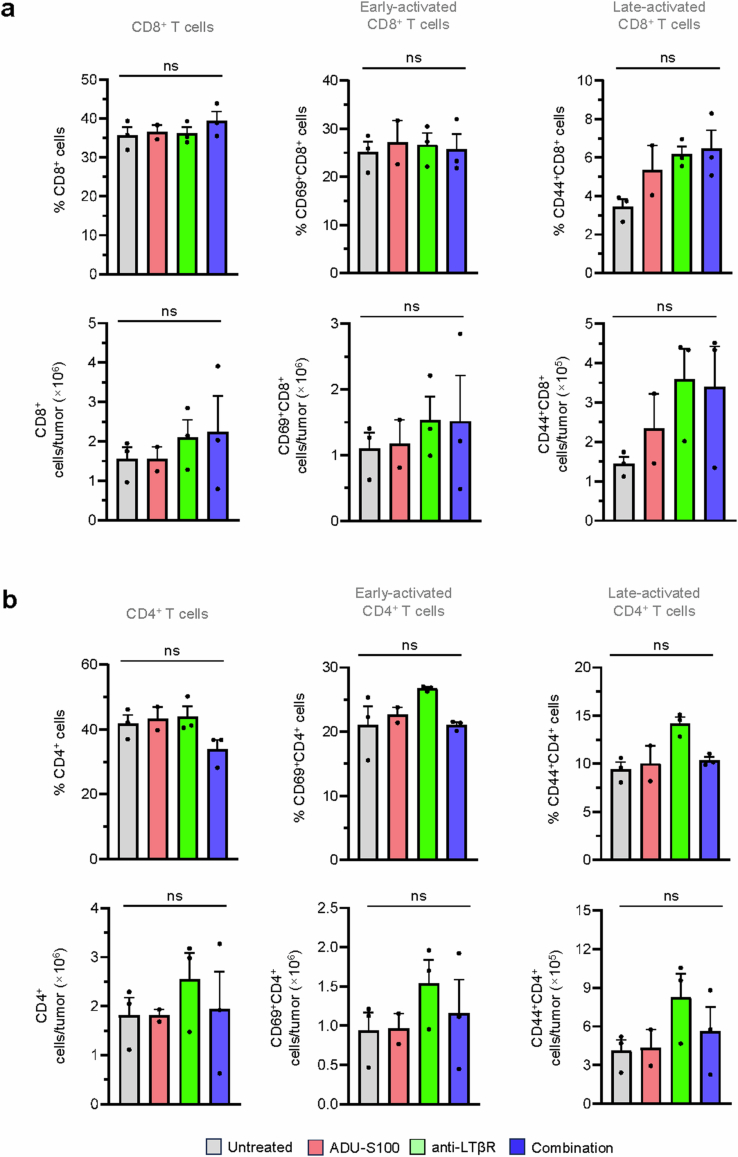
Analysis of tumor-infiltrating T cells. **a**, Upper panels: The abundance of CD8^+^ T cells in primary KPC tumors on the day of tumor resection (Day 14) determined by flow cytometry. Analyses of total CD8^+^ T cells as well as CD69 and CD44 expression were conducted to determine early and late activation states of CD8^+^ T cells, respectively. The abundance of total and activated CD8^+^ T cells is presented as a % fraction of total CD3^+^ T cells. For each data point, three tumors were pooled and dissociated to isolate lymphocytes for FACS analysis. Total 6-9 tumors were examined. Lower panels: The number of total CD8^+^ T cells or activated CD8^+^ T cells per tumor was calculated from the total live cell count after tumor dissociation and the % fractions of these T cells as determined by flow cytometry (Fig. 4a). For each data point, three tumors were pooled, and the obtained cell number was divided by three to yield average T cell count/tumor. Total 6-9 tumors were examined. **b**, A similar analysis of tumor-draining CD4^+^ T cells. A representative result of two independent experiments is shown for each analysis. *p < 0.05; **p < 0.01; ***p < 0.001; ns, not significant.

**Extended Data Fig. 7:**
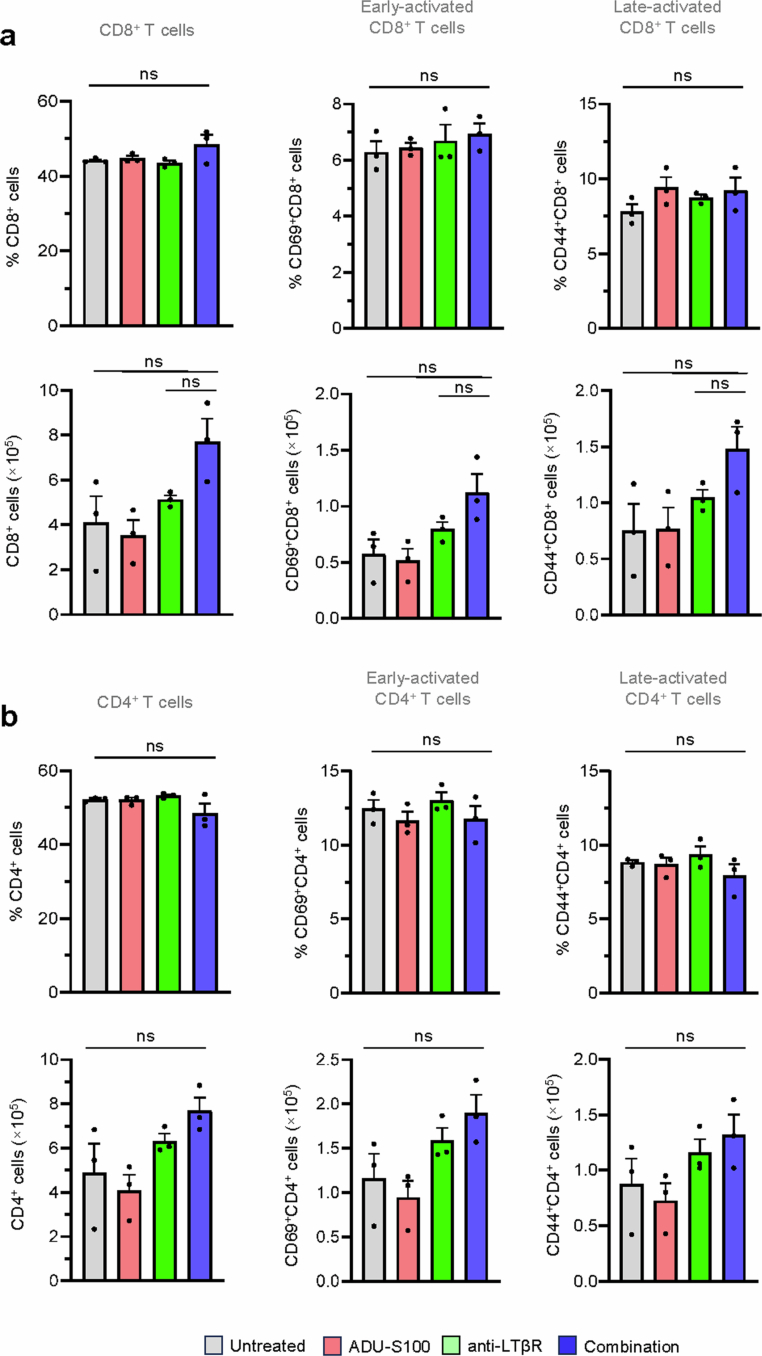
Analysis of T cells in tumor-draining lymph node. **a**, Upper panels: Live lymph node leukocytes were gated for CD3^+^ T cells, and the % fractions of total and activated CD8^+^ T cells in total CD3^+^ T cells were determined by flow cytometry. Lower panels: The numbers of total and activated CD8^+^ T cells in a draining lymph node were calculated from the total leukocyte count in each lymph node and the % fractions of the T cell subsets. For each data point, six draining lymph nodes were pooled and averaged. A total of 18 lymph nodes were examined for each group. A representative of two independent experiments is shown for each analysis. *p < 0.05; ***p < 0.01; **p < 0.001; ns, not significant. **b**, A similar analysis of CD4^+^ T cells in the draining lymph node. Upper panels: The % fractions of total and activated CD4^+^ T cells in total CD3^+^ T cells. Lower panels: The average numbers of total and activated CD4^+^ T cells in a draining lymph node.

Our results suggest that one advantage of combination therapy over LTβR monotherapy is the activation and maturation of tumor-infiltrating B cells. The extensive accumulation of B cells in TLS suggests germinal center-like B cell responses. Immunofluorescence analysis of tumor sections demonstrated considerable accumulation of IgG^+^CD138^+^ B cells within TLS (Fig. 4c), indicating that these B cells were antigen primed, differentiated to antibody-producing plasma cells and have undergone immunoglobulin class switching as expected from the germinal center activities of mature TLS. The presence of CD73^+^ B cells is consistent with the development of memory B cells in these TLS (Fig. 4d). In comparison with B cells, agonist treatments did not significantly increase tumor infiltration of CD4^+^ or CD8^+^ T cells (Extended Data Fig. 6). Likewise, we did not observe significant changes in the T cell fractions in draining lymph nodes (Extended Data Fig. 7).

### Changes in tumor transcriptome upon agonist therapies

**Fig. 6:**
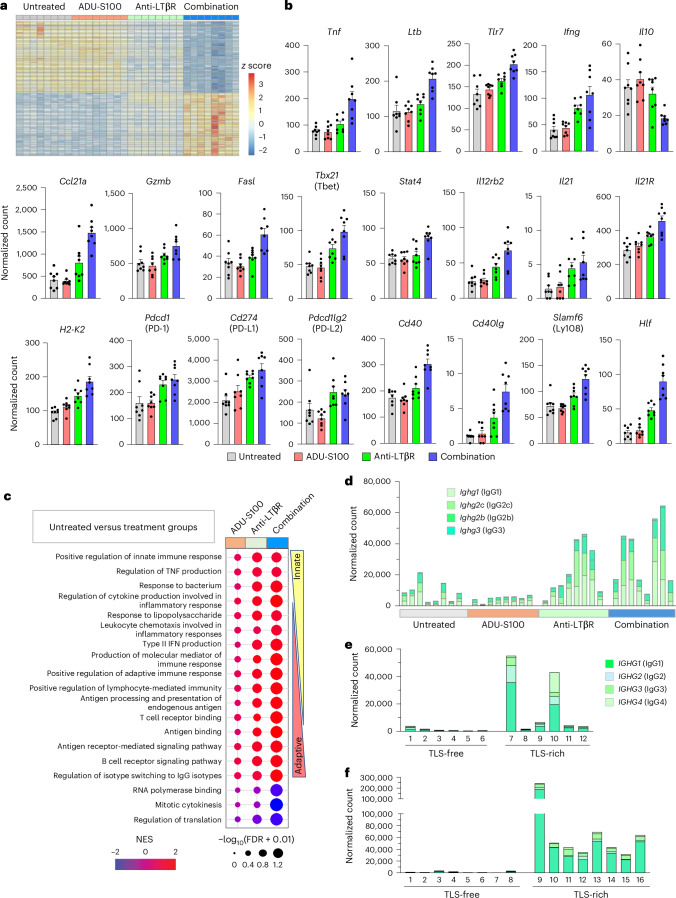
Tumors exhibit a gene expression signature of enhanced immune responses upon agonist combination therapy. Bulk RNA-seq was conducted using total RNA isolated from KPC tumors on the day of tumor resection (day 14), and the data were processed by DEseq2. **a**, Heat map showing differential gene expression between the untreated control and agonist-treated groups. **b**, Expression levels of genes related to inflammation, innate immunity and adaptive immunity in tumors of different treatment groups. One experiment. *N* = 8 tumors were examined representing biological replicates for each group. Data are presented as the mean ± s.e.m. **c**, Gene-set enrichment analysis (GSEA) was performed using the RNA-seq data. Each treatment group was compared to the control group. Normalized enrichment score (NES) for each Gene Ontology term is shown. **d**, Expression of mouse IgG heavy chain detected in tumors. Eight tumors were examined for each group. In these bulk RNA-seq analyses, individual tumors were sequenced without pooling, and the results from two independent experiments were combined. A total of eight tumors were examined per group. **e**, Expression of IgG heavy chain detected in TLS-rich and TLS-free human cancer. Pancreatic ductal adenocarcinomas were categorized into TLS-free and TLS-rich tumors, and the expression of class-switched IgG heavy chain was determined by RNA-seq of these tumors. *N* = 6 tumors from individuals with cancer were examined for each group. **f**, A similar RNA-seq analysis of IgG expression in human breast adenocarcinomas categorized into TLS-free and TLS-rich tumors. *N* = 8 individuals with cancer were examined for each group. FDR, false discovery rate.

**Extended Data Fig. 8:**
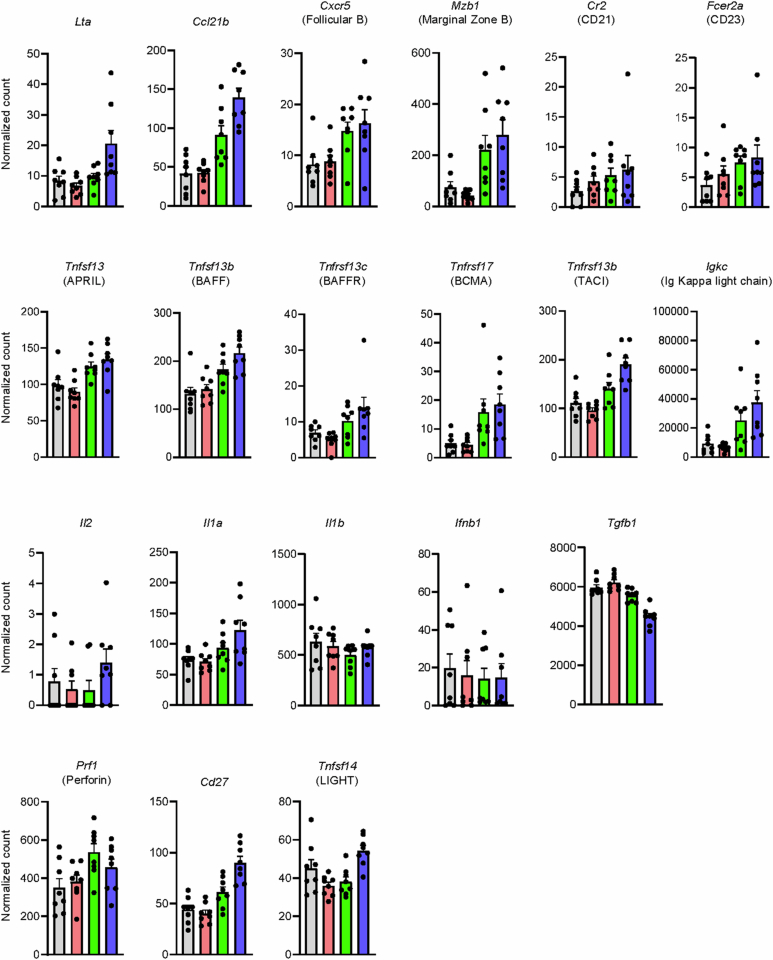
Gene expression signature of enhanced immune responses upon combination therapy. Bulk RNAseq was conducted using total RNA isolated from KPC tumors on the day of tumor resection (Day 14), and the data was processed by DEseq2. The graphs show the expression of different genes related to inflammation, innate immunity, and adaptive immunity in tumors of different treatment groups. One experiment. N = 8 tumors were examined representing biological replicates for each group. Data are presented as the mean ± s.e.m.

We next conducted sequencing of total tumor RNA to characterize the immune landscape of the tumors on the day of tumor resection (day 14). This analysis showed an altered tumor transcriptome in the combination therapy group compared with other groups (Fig. 6a). Among the increased transcripts were those of inflammation/innate immunity-related genes *Tnf*, *Lta*, *Ltb*, *Light* and *Tlr7* (Fig. 6b and Extended Data Fig. 8), many of which were also predicted by our pathway analysis of clinical specimens comparing TLS-rich versus TLS-free breast adenocarcinomas. In addition, the transcripts of effector genes crucial to adaptive immunity, Fas ligand (*Fasl*) and granzyme B (*Gzmb*), as well as a key chemokine for the recruitment of circulating naive T lymphocytes, *Ccl21*, were increased (Fig. 6b). The immunosuppressive cytokine *Il10* was reduced to half of the level of the untreated control. There was a prominent transcriptomic signature for the immunostimulatory type 1 helper T (T_H_1) cell environment in tumors treated with combination therapy. For instance, T_H_1-inducing transcription factor *Tbx21* (Tbet) was increased by twofold. *Il12rb2* and *Stat4* were both significantly increased (Fig. 6b), which is expected to promote T cell differentiation toward the T_H_1 phenotype by enhancing IFNγ expression^37^. Indeed, *Ifng* was significantly elevated in the combination therapy group (Fig. 6b). The significantly increased transcripts of IL-21 and IL-21 receptors (*Il21* and *Il21r*) suggest an active pathway that promotes T_FH_ cell differentiation and IgG production in germinal centers (Fig. 6b)^38^. These two genes were also identified in the pathway analysis of individuals with TLS-rich cancer. A follicular B cell marker *Cxcr5* (the receptor for CXCL13) and the genes necessary for B cell activation by T_FH_ cells in germinal centers (*Cd40*, *Cd40lg*) as well as the plasma cell and memory B cell markers, *Mzb1* and *Cd27*, were significantly increased and consistent with the B cell differentiation associated with the formation of functional TLS (Fig. 6 and Extended Data Fig. 8). Furthermore, B cell survival factors and their receptors important for humoral immunity (*Tnfsf13b*/BAFF, *Tnfsf13*/APRIL, *Tnfrsf17*/BCMA, *Tnfrsf13c*/BAFFR, *Tnfrsf13b*/TACI) were elevated (Extended Data Fig. 8). The effects of LTβR monotherapy were similar but not as robust for many genes.

These findings are consistent with the presence of T_FH_ cells and B cell activation/differentiation in TLS of these tumors as detected by immunostaining (Figs. 1 and 4) and consistent with the findings from flow cytometry (Fig. 4 and Extended Data Fig. 5). There were also increased transcripts of immune checkpoint molecules PD-1 and PD-L1 as well as H2-K2 (major histocompatibility complex class I; Fig. 6b), consistent with an immune responsive tumor microenvironment created by the combination drug treatment. A gene-set enrichment analysis indicated enhanced inflammatory/innate immune responses as well as adaptive immunity and cytotoxicity by combination therapy (Fig. 6c).

We also found increased expression of class-switched IgG immunoglobulin heavy chains in LTβR monotherapy and combination therapy groups (Fig. 6d) reflecting the accumulation of plasma cells and antibody production in TLS (Fig. 4c). These results recapitulated the abundant expression of IgG in TLS-rich, but not in TLS-free, human pancreatic ductal carcinomas and breast adenocarcinomas (Fig. 6e,f).

### Intratumoral B cell and T cell subsets in combination therapy

**Fig. 7:**
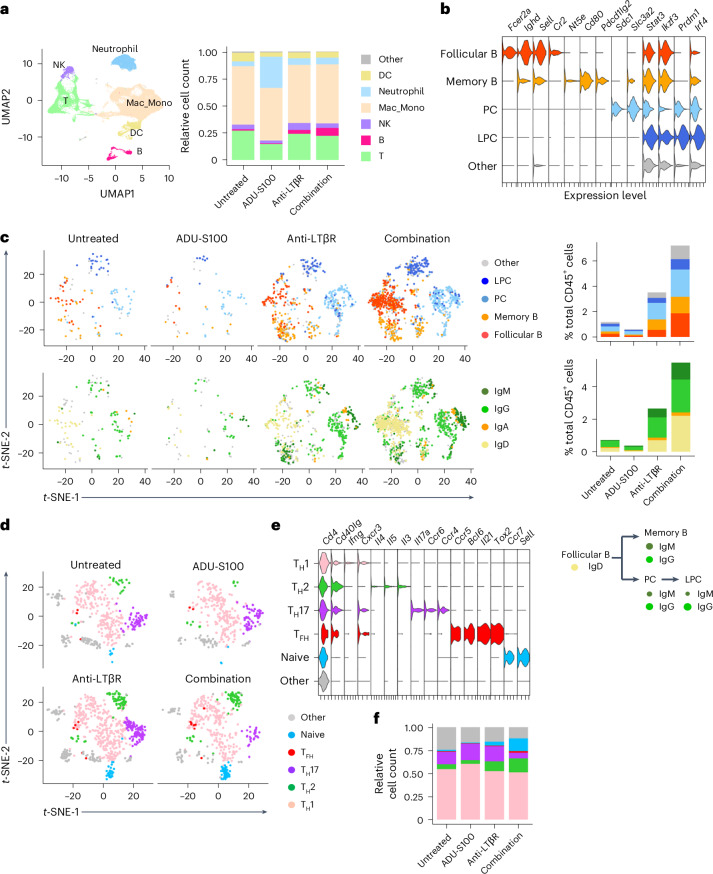
Single-cell RNA-seq analyses of tumor-infiltrating leukocytes. Tumor-infiltrating CD45^+^ cells were analyzed on day 14 by single-cell RNA-seq following FACS sorting. **a**, Left: uniform manifold approximation and projection (UMAP) of different CD45^+^ leukocyte populations was generated using sets of genes for cell-type annotations as listed in Extended Data Fig. 9. Right: relative abundance of different immune cell types is shown as the fractions of total tumor-infiltrating CD45^+^ leukocytes in indicated treatment groups. **b**, Each B cell subtype was identified by expression of indicated marker genes. PC, plasma cell; LPC, long-lived plasma cell. **c**, Upper plots: the abundance of intratumoral B cell subtypes in different treatment groups. Lower plots: the abundance of intratumoral B cell populations expressing different isotypes of immunoglobulins. **d**–**f**, Intratumoral CD4^+^ T cell subtypes (naive, T_H_1, T_H_2 and T_H_17) are shown as separate clusters in the *t-*SNE plots of different treatment groups (**d**). The identity of each T cell subtype was determined by the expression of indicated marker genes (**e**). The relative abundance of CD4^+^ T cell subtypes is shown as the fraction of total intratumoral CD4^+^ T cells in different treatment groups (**f**). DC, dendritic cell.

**Extended Data Fig. 9:**
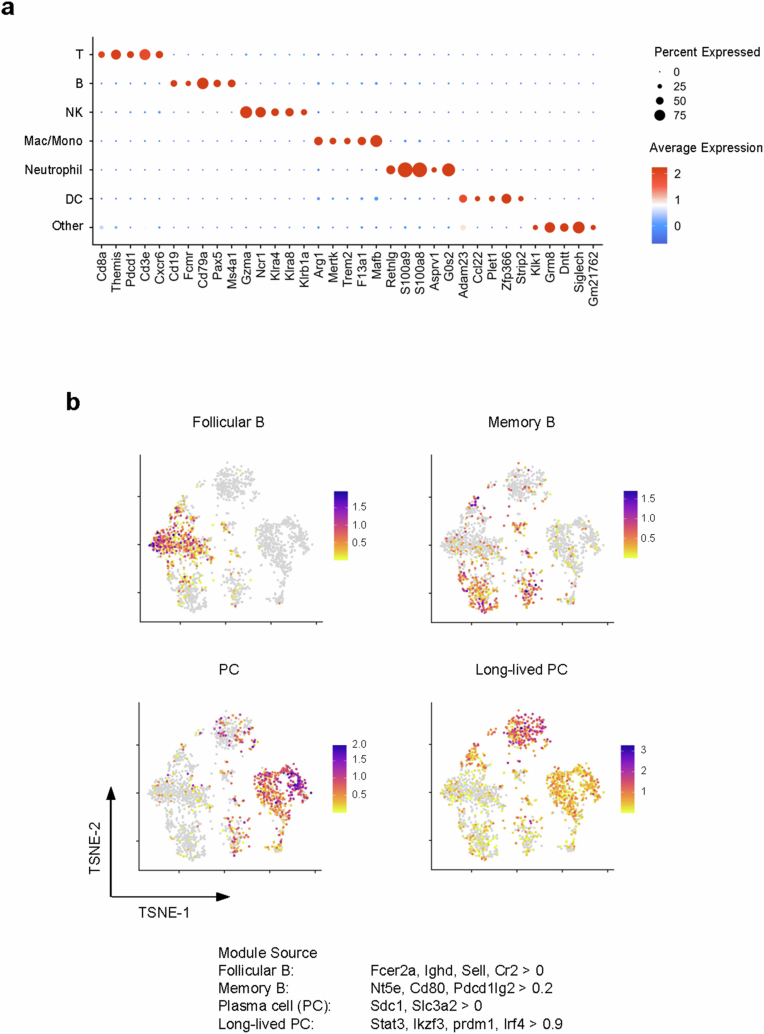
Gene expression patterns used to identify immune cell types in single-cell analysis. **a**, The immune cell types identified in the UMAP clusters shown in Fig. 7 were determined based on the signature gene expression for each cell type. The genes listed here were found among the top 20 genes in each cluster using FindMarkers function of the Seurat application. **b**, Single-cell analysis of B cell subsets. Feature plots with module score for B cell subsets generated from the single-cell RNAseq analysis of tumor-infiltrating CD45^+^ cells on Day 14. Each B cell subset (follicular B, memory B, plasma cell, or long-lived plasma cell) was identified according to indicated marker genes. PC, plasma cell.

The tumor immune environment was further investigated by single-cell RNA-sequencing (RNA-seq) analyses of fluorescence-activated cell sorting (FACS)-sorted tumor-infiltrating CD45^+^ leukocytes on the day of tumor resection. Uniform manifold approximation and projection of different leukocyte subsets showed that the most prominent changes induced by the agonist treatment were the substantial expansions of intratumoral B cells and neutrophils by combination therapy and STING monotherapy, respectively (Fig. 7a and Extended Data Fig. 9a). Combining LTβR agonist with STING therapy canceled the STING-induced neutrophil expansion (Fig. 7a). Further sub-clustering of the B cell population showed that all B cell subtypes, including follicular B cells, memory B cells, plasma cells and long-lived plasma cells^39^, are greatly increased by combination therapy (Fig. 7b,c and Extended Data Fig. 9b). Long-lived plasma cells are terminally differentiated mature B cells that have undergone somatic hypermutations for high-affinity antibody production, and therefore, end products of the germinal center reaction^40^. Our observations support the presence of intratumoral germinal center reactions in this treatment group. Interestingly, there were vast expansions of IgD^+^, IgM^+^ and IgG^+^ B cells, but the expansion of IgA^+^ B cells was limited, suggesting that germinal center B cells of TLS favor class switching to IgG over IgA under combination therapy (Fig. 7c). IgD expression was found exclusively in the follicular B cell population and the expression of class-switched IgG was found in memory B and plasma/long-lived plasma cells (Fig. 7c), again supporting our conclusion for the intratumoral development of mature B cells driven by the germinal center responses taking place within these tumors. The high-affinity IgG-producing long-lived plasma cells could provide long-term protection against future tumor recurrence^40^. LTβR monotherapy had similar but much lesser effects on the expansion of IgG^+^ and total B cells (Fig. 7c). STING activation alone did not expand the intratumoral B cell population or increase immunoglobulin expression, indicating that a substantial TLS formation and antibody production require activation of both STING and LTβR pathways.

**Extended Data Fig. 10:**
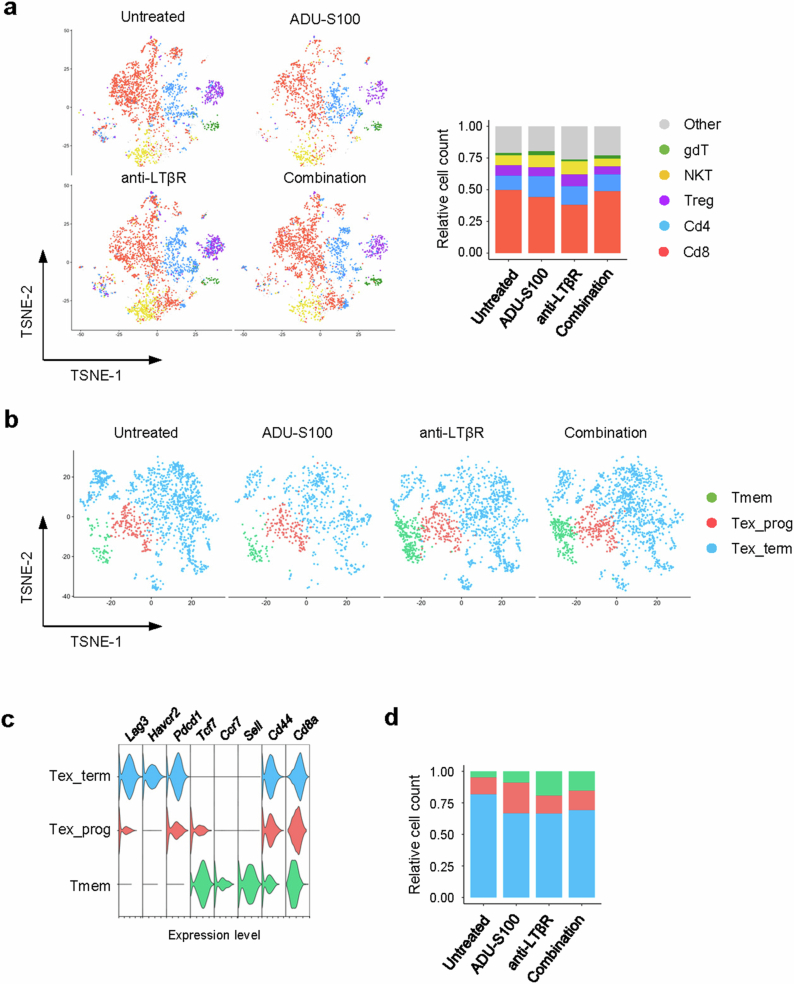
Single-cell RNAseq analysis of tumor infiltrating T cells. **a**, Left, Subclusters of intratumoral CD3^+^ T cells are compared between the treatment groups. T cell subsets are identified in these clusters. Right, Relative abundance of T cell subsets is shown as the fractions of total CD3^+^ T cells. **b**, CD8^+^ T cell population was further subclustered to identify subsets of CD8^+^ T cells infiltrating the tumors. **c**, Subsets of CD8^+^ T cells were identified by the expression of indicated marker genes. T_mem_, memory CD8^+^ T cell; T_ex_prog_, progenitor exhausted CD8^+^ T cell; T_ex_term_, terminally exhausted CD8^+^ T cell. **d**, Relative abundance of different CD8^+^ T cell subsets in total CD8^+^ T cells. Memory CD8^+^ T cell population (green) expanded in treatment groups, especially in LTβR monotherapy and combination therapy groups, compared with the untreated group.

Unlike the prominent effects on B cell expansion, the agonist treatments did not appear to affect the abundance of total CD4^+^, CD8^+^, γδT, natural killer (NK) T or regulatory T cells in tumors (Extended Data Fig. 10a). However, an analysis of CD4^+^ T cell subsets indicated the emergence of a distinct cluster of naive CD4^+^ T cells, identified by *Ccr7* and L-selectin expression (*Cd4*^+^*Ccr7*^hi^*Sell*^hi^), after combination therapy (Fig. 7d–f). CCR7 is a chemokine receptor for CCL19 and CCL21. L-selectin binds to sulfated sialyl Lewis-X carbohydrate structure (MECA-79 epitope) expressed by HEVs to initiate adhesion and extravasation of circulating lymphocytes. The expansion of the naive CD4^+^ T cell population suggests increased recruitment of these lymphocytes from the circulation, facilitated by increased HEV formation in the tumors of this treatment group. Naive CD4^+^ T cells also increased after LTβR monotherapy but to a lesser extent (Fig. 7d–f), possibly corresponding to the formation of fewer HEVs in this group. Furthermore, we found a considerable increase in type 2 helper T (T_H_2) cells in the tumors upon LTβR or combination therapy (Fig. 7d–f). The T_H_2 cytokines expressed by this T cell subset, such as IL-4, IL-5 and IL-13 (Fig. 7e), are important for the immunoglobulin class switching in B cells^41^ and, therefore, important for the maturation of B cells in TLS. In contrast to the naive and T_H_2 populations, the T_H_17 subset of helper T cells diminished considerably by combination therapy (Fig. 7d–f) demonstrating a shift in the T_H_2/T_H_17 balance toward T_H_2 upon combination therapy, likely contributing to the development of humoral immunity in this treatment group^42^.

A subcluster analysis of CD8^+^ T cells identified a cluster of memory CD8^+^ T cells^43–46^ (Extended Data Fig. 10b,c). The relative abundance of memory CD8^+^ T cells increased in treatment groups, especially LTβR monotherapy and combination therapy groups, threefold or more compared with the untreated group (Extended Data Fig. 10b,d). Thus, increased naive CD4^+^ and memory CD8^+^ T cells and expanded T_H_2 and diminished T_H_17 populations were the characteristics of tumor-infiltrating T cells under combination therapy. Overall, the results of our genomic analyses, flow cytometry and immunostaining demonstrated that STING–LTβR combination therapy enhances the adaptive immunity to tumors.

### B cell humoral immunity is essential to neoadjuvant therapy

**Fig. 8:**
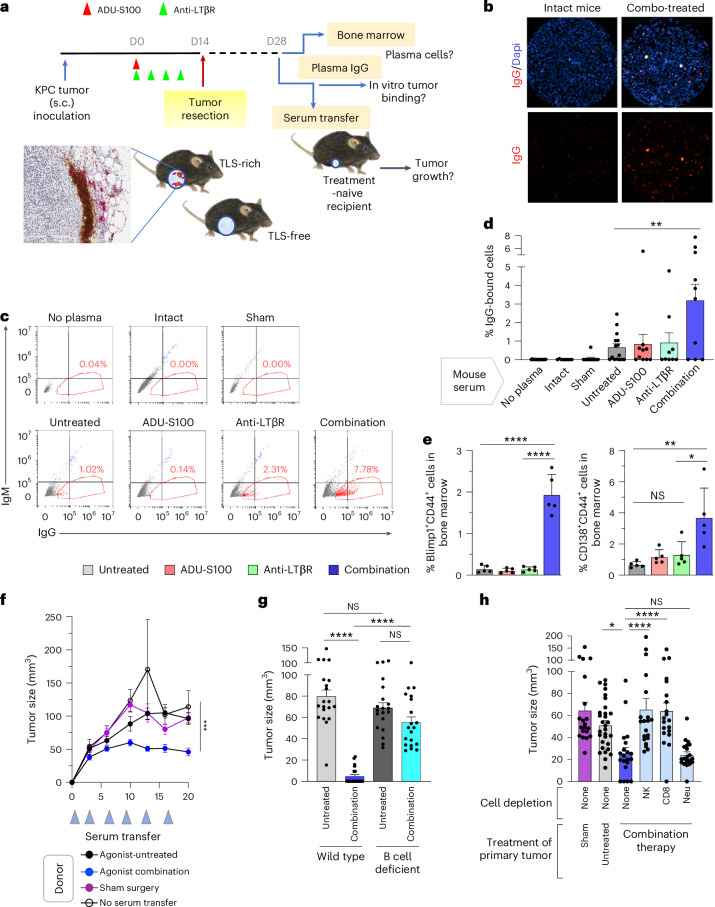
Therapeutic effects of neoadjuvant combination therapy are elicited by cellular and humoral immunity. **a**, Treatment schedule of neoadjuvant agonist therapies for s.c. KPC tumors and blood plasma collection or serum transfer to naive recipients. **b**, Blood plasma was collected 2 weeks after tumor resection, heparinized and diluted at a 1:50 ratio with PBS, and added to cultured KPC cells. The binding of plasma IgG to KPC cells was visualized by fluorescently labeled sheep anti-mouse IgG (red). Scale bar, 1 mm. **c**, Similarly, blood plasma collected from different mouse groups was incubated with KPC cells in single-cell suspension, and the binding of plasma IgG to these cells was detected by the right shift of the cell population in FACS analysis (indicated by red gating). IgM binding was undetectable compared with the sham and intact mouse (non-immune plasma) controls in this assay. **d**, Percentage fraction of IgG-bound KPC cells in total KPC cells. Plasma samples from 10–14 different mice from each group were examined for KPC cell binding. A representative result of three experiments. **P < 0.01. **e**, Bone marrow was collected from the left and right femurs and tibias of each agonist-treated or untreated mouse 2 weeks after tumor resection, and bone marrow leukocytes were analyzed by flow cytometry using Blimp1 and CD44 staining as markers for long-lived plasma cells. One experiment. *N* = 5 per group. *P < 0.05, **P < 0.01, ****P < 0.0001. **f**, Blood serum was collected from different donor mouse groups (sham surgery, agonist untreated, combination therapy treated) and transferred to naive recipient mice several times after tumor inoculation. The tumor growth in these animals was monitored for 20 days. One experiment. *N* = 20 tumors were analyzed for each serum transferred group. *N* = 10 for controls receiving no serum transfer. ***P < 0.001. **g**, Neoadjuvant treatment and tumor resection followed by tumor reinoculation were carried out in wild-type or B cell-deficient CD79a knockout mice as described in Fig. 3e. Tumor size was compared between different mouse groups on day 9 after tumor reinoculation. The results of two independent experiments were combined. *N* = 20 tumors were analyzed for each group. ****P < 0.0001. **h**, Neoadjuvant treatment and tumor resection followed by tumor reinoculation were carried out in wild-type mice as described in Fig. 3e. Starting from one day before the reinoculation of KPC tumors, anti-NK1.1, anti-CD8 or anti-Ly6G was administered (i.p.) to deplete NK cells, CD8^+^ T cells or neutrophils, respectively. Tumor size was compared between different mouse groups on day 6 after tumor reinoculation. One experiment. *N* = 20 tumors were analyzed for each group. *P < 0.05, ****P < 0.0001. Data are presented as the mean ± s.e.m. and analyzed using one-way (**d**, **e**, **g** and **h**) or two-way (**f**) ANOVA with Tukey’s test for statistical significance. All replicates represent biological replicates. Source data

We showed that both LTβR monotherapy and STING–LTβR combination therapy induced TLS in primary KPC tumors; however, only combination therapy significantly increased activated B cells and memory B cells in the tumors, and combination therapy provided much stronger protection against the second tumor challenge than LTβR monotherapy. These results may reflect qualitative differences between TLS (or ‘fitness’ of TLS) of the two treatment groups and suggest the importance of TLS-driven humoral immunity enhanced by the combination therapy. Therefore, we measured antibodies in the blood of these mice with specificity for tumor cells. Blood plasma was collected from the agonist-treated or untreated mice 2 weeks after the tumor resection (Fig. 8a), diluted at a 1:50 ratio in PBS and incubated in vitro with live KPC cells. The binding of plasma IgG and IgM to the tumor cell surface was determined by flow cytometry using fluorescently labeled goat anti-mouse IgG and IgM antibodies. This study showed that the IgG-bound KPC cells were significantly increased in the combination therapy group, suggesting high-titer and/or high-affinity antitumor IgG productions by B cells of this group (Fig. 8b–d). Specific binding of IgM was undetected in this assay. Clinical studies have shown that patients with cancer exhibiting IgG-bound tumor cells have considerable therapeutic responses to immune checkpoint inhibition and prolonged progression-free survival^47^, signifying the importance of our findings. Our results suggest the presence of antibody-secreting plasma cells residing in the bone marrow of combination therapy-treated mice. Therefore, we analyzed the bone marrow 2 weeks after tumor resection (Fig. 8a). Flow cytometry analysis demonstrated marked increases in Blimp1^+^CD44^+^ cells and CD138^+^CD44^+^ cells in the combination therapy group, but not in other groups, suggesting accumulation of long-lived plasma cells in the bone marrow of these mice (Fig. 8e).

A serum transfer experiment further demonstrated the importance of humoral immunity. We collected serum from mice 2 weeks after the neoadjuvant agonist combination therapy and tumor resection surgery (Fig. 8a). The serum was pooled for each group, and heat inactivation to abolish complement activity was carried out. The serum was aliquoted, and 100 μl was intraperitoneally given to treatment-naive recipient mice on the day of KPC tumor injection to these mice. These mice continued to receive serum transfer two times per week, and tumor growth was monitored. In this study, the mice receiving serum transfer from the donors that underwent tumor resection surgery without neoadjuvant therapy showed a tumor growth rate similar to that of the control mice receiving no serum transfer or serum transfer from healthy mice with sham surgery (Fig. 8f). In comparison, the mice receiving serum transfer from the donors that underwent neoadjuvant combination therapy before the surgery showed significant suppression of tumor growth, demonstrating the presence of antitumor immunity in the serum of neoadjuvant-treated mice (Fig. 8f). To further demonstrate the importance of humoral immunity, we treated B cell-deficient CD79a knockout mice with neoadjuvant combination therapy followed by the tumor rechallenge 2 weeks later. Unlike wild-type mice, these mice failed to reject the reinoculated tumors (Fig. 8g), indicating the essential role of B cells in the therapy-induced development of antitumor immunity. The combined results demonstrate the importance of B cell humoral immunity.

### Cell-mediated immunity is required for neoadjuvant therapy

We next examined the role of cell-mediated immunity in the therapeutic effect of the neoadjuvant agonists. KPC tumor-bearing mice were treated with or without neoadjuvant STING–LTβR combination therapy followed by tumor resection surgery. Two weeks later, starting from one day before reinoculation of KPC cells, these mice received intraperitoneal injection of anti-CD8, NK1.1 or Ly6G antibodies to deplete CD8^+^ T cells, NK cells or neutrophils, respectively. In this study, neoadjuvant-treated mice lost the ability to inhibit the growth of reinoculated tumors upon deletion of CD8^+^ T cells or NK cells but not neutrophils (Fig. 8h). These results demonstrate that cellular immunity by CD8^+^ T cells and NK cells is also crucial for the antitumor immunity acquired through neoadjuvant combination therapy.

## Discussion

In this study, we demonstrated that simultaneous activation of innate immune effectors, STING and LTβR, induces strong B cell-mediated and T cell-mediated immunity against tumors. The immediate-early response to this treatment was mediated mainly by CD8^+^ T cells. The delayed but prolonged response was mediated by humoral immunity of B cells, which, together with the cellular immunity of CD8^+^ T cells and NK cells, exerted a lasting antitumor effect. The initial response by CD8^+^ T cells allows adaptive immunity to promptly engage in tumor inhibition upon initiation of the treatment. On the other hand, prolonged response is important for sustained treatment benefits, providing protection against future tumor recurrence. Tumor recurrence is a common cause of cancer death in patients who received drug treatment and surgery; therefore, this mechanism has an important clinical implication.

We demonstrated that mature functional TLS with germinal center reactions can be therapeutically induced in TLS-free tumors. T_FH_ cells were found in these TLS closely interacting with B cells, and these B cells expanded and differentiated to memory and IgG-producing plasma cells in the ectopic lymphoid structures developed in tumors. LTβR activation promoted HEV and TLS formation, but an additional contribution from STING was necessary for the full responses of TLS-associated B cells and the development of strong adaptive immunity against tumors. When used as neoadjuvant therapy, the STING and LTβR agonist combination effectively immunized mice against the second tumor challenge, leading to long-term survival. STING activation may also improve the fitness of TLS developed by a treatment with LIGHT or CXCL13, the effectors of the LTβR pathway^25,28,48^. Our findings provide a foundation for drug induction of TLS for cancer immunotherapy.

TLS formation is a prominent outcome of this treatment. However, we cannot rule out the possibility that tumor-draining lymph nodes are mainly responsible for the observed immune responses against tumors. We observed a trend of a moderate increase in total lymphocyte counts in draining lymph nodes indicating that these lymph nodes are affected by the treatment. However, the fractions of activated B cells and T cells in these lymph nodes were not significantly different between the three different treatment groups, raising the question of how lymphocytes in lymph nodes contributed to the response to the combination therapy. Instead, our findings suggest that the fitness of TLS greatly influenced the development of long-term antitumor immunity. There was a robust increase in B cell activation and differentiation in TLS, but not in the draining lymph nodes, upon combination therapy compared with monotherapies, and this increase was associated with a marked accumulation of Blimp1^+^ long-lived plasma cells in the bone marrow. Furthermore, although there were no notable changes in lymph node germinal centers, we found a significant increase in the serum level of tumor cell-targeting IgG in the mice of the combination therapy group. STING or LTβR monotherapy did not produce these beneficial effects. It is known that TNF and IFNγ promote B cells to undergo immunoglobulin class switching to IgG, while TGFβ promotes class switching to IgA^39,49^. Our single-cell analysis showed that TLS-B cells favor class switching to IgG over IgA under combination therapy. This finding is consistent with the elevated TNF and IFNγ and reduced TGFβ in the tumor microenvironment created by this treatment. Overall, these findings support the idea that TLS play an important role in the antitumor immunity potentiated by neoadjuvant combination therapy. TLS spontaneously developed in human lung adenocarcinomas or mouse KPAR lung tumors produce antibodies against endogenous retrovirus envelope glycoproteins^50^. The identification of the target tumor antigens for antibodies generated by the drug-induced TLS awaits further investigation.

Our study showed that B cells and T cells are both essential for the development of TLS and HEVs. Interestingly, CD4^+^ T cells were required for the formation of TLS but dispensable for HEVs. B cells and CD8^+^ T cells were required for TLS and HEV formations. These findings suggest that T cells not only are the first responder to combination therapy directly targeting tumor cells but also act critically as the architects of the tumor immune landscape suitable for B cell infiltration and expansion, leading to the formation of mature TLS, supporting the development of humoral immunity against cancer. On the other hand, therapy-induced TLS may facilitate the development of T cell immunity, creating a feedback loop mechanism for the synergy between antitumor B cells and T cells. TLS are thought to provide hubs for T cell activation through antigen presentation and cytokine productions by B cells^6,8^. In addition, recent studies suggest that TLS create a supportive microenvironment for TCF1^+^ T cells to preserve their stem-like states, allowing the development of sustained T cell immunity against tumors^51,52^. These T cell-supporting properties of TLS may explain how neoadjuvant combination therapy produces effective cellular immunity against tumors. Likewise, tumor-associated HEVs create active entry sites and perivascular niches for TCF1^+^ stem-like T cells, which is implicated in better clinical responses and survival of patients with melanoma treated with immune checkpoint inhibition therapies^26,27^. HEVs are an integral component of the TLS-rich microenvironment, along with B cell germinal centers and T cell subsets localized within and around TLS.

Unlike other reported strategies of TLS induction, TLS induction by this method does not require viral antigens, viral infection or artificial genetic manipulations to boost immune response, thus making it feasible for clinical applications in cancer treatment. Using this approach, functional TLS can be induced without any preconditioning, in different tumor types and anatomical sites, not restricted to certain organs such as the immune environment of the lung^50,53,54^—TLS developed in adenocarcinomas and sarcomas in the pancreas, mammary gland, skeletal muscle and subcutaneous tissues using our drug treatment strategy. The long-term survival enabled by this treatment recapitulates improved survival of patients with cancer who exhibited tumor-associated TLS^5,7–10^. The high-affinity antibody-producing long-lived plasma cells and memory B cells generated in these TLS could provide lifelong protection against recurrent tumors^40^. The effective tumor immunization by this strategy is expected to improve the efficacy of immune checkpoint blockade, cancer vaccines and other cancer immunotherapies. Thus, our study suggests broad therapeutic applications in cancer. Since STING agonists are clinically available^55,56^ and humanized agonistic monoclonal antibodies to LTβR can be developed, this strategy is readily translatable to clinical use for cancer treatment.

## Methods

### Clinical cancer specimens

Informed written consent was obtained from all participants involved in the study, and all clinical investigations were conducted in line with the principles of the Declaration of Helsinki. Formalin-fixed paraffin-embedded tissue samples of pancreatic or breast cancer were collected at the National Cancer Center Hospital, Japan. The use of the cancer specimens was approved by the Institutional Review Board (protocol 2005-077). Specimens were from individuals who underwent macroscopic curative resection. None of the individuals had received any therapy before surgery. All cases of pancreatic cancer were conventional pancreatic ductal carcinomas; cases of adenocarcinomas originating in intraductal papillary mucinous neoplasms or mucinous cystic neoplasms were excluded, as were secondary tumors and post-neoadjuvant cases. Cases with autoimmune pancreatitis-associated cancers were excluded. Breast cancer specimens (stage I–III, invasive ductal adenocarcinoma) were described previously^34^.

### Mice

The procedures involving animals used in this study were approved by the Institutional Animal Care and Use Committee of Johns Hopkins University. C57BL/6NHsd mice were purchased from Inotiv (Envigo) and used at 8–10 weeks old for the study. Female mice were used for the orthotopic mammary tumor and rhabdomyosarcoma models, and male mice were used for the subcutaneous and orthotopic pancreatic tumor models. Nude mice on the C57BL/6 background (B6.Cg-*Foxn1*^*nu*^/J) were obtained from The Jackson Laboratory (strain number 000819). B cell-deficient *Cd79a*-Cre homozygous C57BL/6 mice (B6.C(Cg)-Cd79a^tm1(cre)Reth^/EhobJ) were obtained from the Jackson Laboratory (strain number 020505).

### Cell lines

The MMTV-PyMT mouse mammary tumor-derived Py230 cells were purchased from the American Type Culture Collection and cultured in F-12K medium with 5% fetal bovine serum, penicillin–streptomycin and 0.1% MITO+ serum extender. The *Kras*^*LSL.G12D/+*^*Trp53*^*LSL.R172H/+*^*Pdx1*-Cre mouse-derived pancreatic tumor KxPxCx cells (KPC) were a gift from E. Jaffee of Johns Hopkins University^35^ and cultured in RPMI 1640 medium supplemented with 10% FBS, 1% MEM non-essential amino acid solution, 1 mM sodium pyruvate and 50 U ml^−1^ penicillin–streptomycin. Mouse rhabdomyosarcoma 76-9 cells were a gift from R. Kaplan of the National Cancer Institute and cultured in RPMI 1640 medium supplemented with 10% FBS, 50 mM 2-mercaptoethanol and 50 U ml^−1^ penicillin–streptomycin.

### Mouse tumor models

KPC tumor cells were subcutaneously injected into the flanks of syngeneic male mice at 3 × 10^6^ cells. When the tumor size reached approximately 100 mm^3^ 10 days later, randomized mice received STING agonist ADU-S100 (MCE MedChemExpress) alone, anti-LTβR antibody 4H8 (Adipogen) alone, or both agonists. ADU-S100 was administered once via intratumoral injection at 2 μg per tumor (day 0), and anti-LTβR was administered intraperitoneally at 100 μg every 3–4 days, for a total of four times until day 10 (ref. ^24^). Tumors were surgically resected on day 14. For the orthotopic mammary tumor model, Py230 cells were suspended in 50% Matrigel and orthotopically injected into the mammary fat pad at 1 × 10^6^ cells per injection. Thirty-two days later, the mammary tumors reached approximately 200 mm^3^ and received the agonist treatment as described above. For the orthotopic rhabdomyosarcoma model, 76-9 cells were injected at 1 × 10^5^ cells intramuscularly to the calf muscle in the area adjacent to the tibia. The tumors reached approximately 100 mm^3^ in 7 days and received agonist treatment. For the survival study using the tumor recurrence model, mice that died due to the failure in complete tumor resection were excluded from the analyses. All other data points were included. Data collections and analyses were not performed blind to the conditions of the experiments.

### Immunostaining

KPC tumors were dissected, embedded in OCT compound and rapidly frozen in liquid nitrogen. Cryosections of 7 µm or 10 µm in thickness were obtained using a cryostat and subjected to immunostaining. First, sections were fixed with 4% formaldehyde for 10 min at 24 °C. Permeabilization was performed using 0.1% Triton X-100 in PBS for 10 min, followed by blocking with a 1% BSA–PBS solution to reduce nonspecific binding. For colorimetric immunohistochemistry, BloxAll (Vector Laboratories) was used to block endogenous peroxidase and alkaline phosphatase. After blocking, sections were washed twice with PBS and incubated with primary antibodies overnight at 4 °C, as follows: (1) B cell and T cell markers: rat anti-mouse CD19 Alexa 647 conjugate, clone 1D3, BD Pharmingen, 557684; rat anti-mouse CD4 Alexa 488, clone GK1.5, BioLegend, 100424; (2) immune and endothelial cell markers: rat anti-mouse CD73, clone TY/11.8, BioLegend, 127202; PE-conjugated rat anti-mouse CD138 (Syndecan-1), clone 281.2, BioLegend, 142504; Armenian hamster anti-mouse CD69, clone H1.2F3, BioLegend, 104516; Rat anti-mouse F4/80 Alexa 488, clone BM8, eBioscience, 53-4801-82; rat anti-mouse interferon-β monoclonal antibody, clone RMMB-1, PBL Assay Science, 22400-1; rat anti-HEV marker monoclonal antibody Alexa 488, clone MECA-79, eBioscience, 53-6036-82; Rabbit anti-CD31, Abcam, ab28364; and (3) rabbit anti-CD23, Invitrogen, PA5-79242; goat anti-mouse IgG-Alexa 488, Invitrogen, A11001; rabbit anti-Bcl6, Abcam, ab272859 and Rabbit anti-Ki-67, GeneTex, GTX16667. The following day, sections were washed three times with PBS, and when necessary, incubated with appropriate secondary antibodies for 1 h at 24 °C. Alexa-conjugated secondary antibodies were purchased from Invitrogen. Then sections were washed three times with PBS, and nuclei were counterstained with DAPI for 10 min. Finally, sections were washed twice with PBS and mounted for imaging. Stained slides were scanned by Aperio Versa (Leica) for Halo image analyses (Indica Labs) using the Cytonuclear module. The tumor area (mm^2^) of each scanned section was used to determine the densities of TLS, HEVs and lymphocytes.

### In vivo time-course study of STING activation in KPC tumors

To assess the activation of the STING pathway in the tumor microenvironment, three time points were established: 2 h, 4 h and 48 h. Mice bearing KPC tumors were intratumorally injected with 2 µg of a STING agonist ADU-S100, with injections staggered at 10-min intervals between mice to ensure precise timing for endpoint collection. At each time point, mice were euthanized via CO_2_ exposure followed by cervical dislocation.

### Western blot analysis of tumor samples

Tumor samples were dissected and immediately snap-frozen in liquid nitrogen. The frozen tumors were thoroughly homogenized on ice in a lysis buffer containing 1× protease and 1× phosphatase inhibitors (Sigma-Aldrich), 10 nM okadaic acid and 0.5 mM phenylmethylsulfonyl fluoride, using a mechanical homogenizer to ensure complete cell lysis. To further disrupt cells and solubilize proteins, the homogenates were sonicated. The samples were then incubated at 4 °C for 1 h with gentle agitation to enhance maceration. Following incubation, the homogenates were centrifuged at 10,000*g* for 15 min at 4 °C to pellet cellular debris. The resulting supernatant was carefully transferred to clean tubes, and centrifugation was repeated twice to ensure sample purity. Protein concentrations were quantified using the bicinchoninic acid assay (Thermo Scientific). Protein extracts (25 µg per sample) were resolved by SDS–PAGE using 4–15% polyacrylamide gels (Bio-Rad) and subsequently transferred to polyvinylidene fluoride membranes (Bio-Rad). Membranes were blocked for 1 h at room temperature with a 3% BSA solution in Tris-buffered saline containing 0.1% Tween-20 (TBS-T). After blocking, the membranes were incubated overnight at 4 °C with primary antibodies diluted in 3% BSA–TBS-T. The following primary antibodies were used: phospho-IRF-3 (Ser396) (4D4G) rabbit antibody (Cell Signaling Technology); IRF3 polyclonal antibody (Invitrogen); IFNβ1 (D2J1D) rabbit antibody (Cell Signaling Technology); β-actin mouse monoclonal antibody (Sigma-Aldrich). After primary antibody incubation, membranes were washed and incubated with horseradish peroxidase-conjugated secondary antibodies for 1 h at 24 °C. Antibody binding was visualized using the Clarity Western ECL substrate (Bio-Rad). β-actin was used as the internal loading control to ensure equal protein loading. Protein bands were imaged using the ChemiDoc MP Imaging System (Bio-Rad), and densitometric analyses were performed using ImageJ software (National Institutes of Health).

### In vitro time-course study of STING activation in KPC and HUVEC cells

To evaluate the activation of the STING pathway in tumor cells (KPC) or endothelial cells (human umbilical vein endothelial cells (HUVECs)) in vitro, seven time points were established: 0, 30, 60, 90, 120, 180 and 300 min. KPC cells were cultured to confluence in 3.5-cm dishes using RPMI GlutaMAX medium (61870-036, Gibco). HUVEC cells were cultured to confluence in endothelial cell growth media (CCM027, R&D Systems). Before treatment, cells were washed twice with 1× PBS to remove residual media. Fresh medium containing either 10 μM STING agonist ADU-S100 or PBS (vehicle control) was then added to the cells. At each endpoint, cells were washed twice with 1× PBS and immediately preserved at −80 °C for subsequent analysis.

### Western blot analysis of KPC and HUVEC cells

Cells were harvested using a RIPA buffer supplemented with 0.3% SDS, 1× protease and 1× phosphatase inhibitors (Sigma-Aldrich) on ice. The cell homogenates were vortexed three times for 30 s each and sonicated twice at 10% amplitude, with 30-s intervals on ice between each sonication. Then, samples were centrifuged at 10,000*g* for 15 min at 4 °C to pellet cellular debris. The supernatant was collected, and protein concentrations were determined using the bicinchoninic acid assay. For protein analysis, 10 µg of total protein was resolved and transferred to polyvinylidene fluoride membranes, blocked and immunoblotted, and signals were detected as previously described for tumor samples. Protein bands were visualized using X-ray films (34090, Thermo Scientific). The resulting images were scanned and quantified using ImageJ. β-actin was used as the internal loading control to ensure equal protein loading.

### Flow cytometry

Tumor-infiltrating lymphocytes and lymphocytes of the draining lymph nodes were analyzed by FACS on day 14, on the day of tumor resection. Tumors were excised and cut into small fragments with sterilized scissors in 0.5–1.0 ml of RPMI containing Liberase (300 µg ml^−1^). After adding 3.0–3.5 ml more Liberase-containing RPMI, the tumor fragments underwent enzymatic digestion and mechanical dissociation using the GentleMACS Octo dissociator (protocol: hard tumor dissociation, TDK-3, 1 h at 37 °C). Following dissociation, the cell suspension was filtered using a 70-µm strainer and then washed twice with RPMI medium at 400 revolutions per minute for 5 min. For flow cytometry analysis, live leukocytes were stained with the following markers: CD19 (APC), CD44 (BV785), CD69 (AF488), CD138 (PE), CD73 (FITC), PD-L2 (BV421), CD3 (AF647), CD4 (BV421), CD8 (AF488), CD62L (APC) and Ghost Dye (V510). Stained cells were analyzed with CytoFLEX LX Flow Cytometer (Beckman Coulter). Flow cytometry data were analyzed with CytExpert Analysis Software version 2.5.0.77 (Beckman Coulter).

Analysis of STING activation in leukocytes was conducted by flow cytometry as follows: Leukocytes were isolated from the peripheral blood, peritoneum, spleen or lymph nodes of normal mice and incubated in vitro with 7.2 μg ml^−1^ STING agonist ADU-S100 (dissolved in PBS) or PBS (control) for 2 h at 37 °C. Phosphorylation of IRF3 (Ser385) in CD11b⁺ macrophages/monocytes, CD3⁺ T cells, CD19⁺ B cells and NK1.1⁺ NK cells was assessed using flow cytometry. Cells were first stained with surface markers CD11b (BV610), CD3 (PerCP-Cy5.5), CD19 (APC), NK1.1 (PE) and Ghost Dye (BV510). They were then permeabilized using the eBioscience Transcription Factor Staining Buffer Set (Thermo Fisher) before intracellular staining with α-phospho-IRF3 (FITC).

### Mouse tumor RNA-seq

Subcutaneous KPC tumors were collected 14 days after agonist treatment started and homogenized using Precellys lysing kits CK28 in Precellys Evolution Touch homogenizer with Cryolys Evolution (Bertin Technologies SAS). The total RNA was isolated from tumors using TRIzol (Thermo Fisher Scientific), and a poly(A) RNA-seq library was prepared following Illumina’s TruSeq-stranded-mRNA sample preparation protocol. RNA integrity was verified with Agilent Technologies 2100 Bioanalyzer. Poly(A) tail-containing mRNAs were purified using oligo-(dT) magnetic beads with two rounds of purification. After purification, poly(A) RNA was fragmented using divalent cation buffer at an elevated temperature. Quality-control analysis and quantification of the sequencing library were performed using Agilent Technologies 2100 Bioanalyzer High Sensitivity DNA Chip. Paired-ended sequencing was performed on Illumina’s NovaSeq 6000 sequencing system.

After the removal of the reads that contained adaptor contamination, low-quality bases and undetermined bases by Cutadapt, sequence quality was verified using FastQC. HISAT2 was used to map reads to the genome of ftp://ftp.ensembl.org/pub/release-101/fasta/mus_musculus/dna/, and StringTie was used for assembly. To estimate the expression levels of all transcripts, StringTie and ballgrwn were used. The mRNA differential expression analysis was performed using the R package DESeq2 between two different groups (and by R package edgeR between two samples). The mRNAs with a false discovery rate below 0.05 and absolute fold change ≥ 2 were considered differentially expressed mRNAs. The heat map was generated with the top 100 genes exhibiting the most significant false discovery rate in DESeq2 analysis. *z*-scores of the normalized value were applied to the pheatmap package (version 1.0.12).

### Single-cell RNA-seq

Single-cell RNA-seq was performed in two independent mouse experiments. KPC tumors were harvested from mice on day 14. In each experiment, ten tumors were pooled for each treatment group and dissociated as described above for flow cytometry using Liberase and GentleMACS Octo dissociator. After dead/live cell staining and blocking nonspecific binding using anti-FcRII or anti-FcRIII (BioLegend) for 10 min, cells were stained with anti-CD45 for 1 h at 4 °C. Live CD45^+^ leukocytes were sorted by CytoFLEX SRT (Beckman) for single-cell library generation using 10x Chromium Next GEM Single Cell 5′ Kit v2, 4, followed by sequencing using Illumina NovaSeq (Illumina). The FASTQ files were uploaded into 10x Genomics Cloud CLI (10x Genomics) for alignment and mapping using Cell Ranger Count v7.1.0 with Mouse (mm10) as the reference. The various functions in the Seurat package (version 5.0.1) for the R statistical software (v.4.3.2; http://www.R-project.org/) were used to analyze the single-cell RNA-seq data. Cells expressing <300 genes or >5,000 genes and cells whose mitochondrial content >6% were excluded.

### RNA-seq of clinical cancer specimens

Five to ten 7-µm-thick formalin-fixed paraffin-embedded tissue sections were used for RNA isolation from each sample. RNA-seq was carried out by Azenta. For pancreatic cancer specimens, a clinical pathologist removed sentinel lymph nodes and intestinal tissues from each section by scraping under a dissection microscope using a H&E-stained serial section to guide tissue removal. PertekFlow was used for trimming, quality control, alignment by STAR (2.7.8a), annotation with hg38_ensembl_release91_v2 and differential analysis with DESeq2.

### Detection of plasma immunoglobin binding to tumor cells

Cultured KPC cells were dissociated by 0.05% Trypsin-EDTA for 3 min and neutralized with 10% FBS in PBS. Cells were stained with LIVE/DEAD Cell staining kit (Themo Fisher Scientific) for 10 min at 4 °C and washed and treated with 10% goat serum/PBS for 10 min at 4 °C. The total of 2 × 10^5^ cells were then suspended in 30 μl of 1:50 diluted mouse blood plasma collected 2 weeks after the tumor resection surgery and incubated for 1 h at 4 °C. Cells were washed with FACS buffer and incubated with 50 μl of 1:1,000 diluted goat anti-mouse IgG-Alexa 647 and anti-mouse IgM-Alexa 488 for 30 min at 4 °C. Cells were washed, fixed and washed again for flow cytometry to quantitatively determine the mouse IgM and IgG binding to the KPC cell surface.

### Serum transfer

Mouse blood serum was collected from 19 mice 2 weeks after neoadjuvant agonist therapy and tumor resection surgery. The serum was pooled, and heat inactivation to abolish complement activity was carried out at 56 °C for 10 min. The serum was aliquoted, and 100 μl was intraperitoneally given to treatment-naive recipient mice on the day of KPC tumor injection to these mice. The mice continued to receive serum transfer two times per week, and the tumor growth was monitored.

### Immune cell depletion

For T cell depletion experiments, mice were injected intraperitoneally with 200 μg of anti-mouse CD4 (clone GK1.5, BioXCell BE0003-1) and/or CD8 (clone 2.43, BioXCell BE0061) monoclonal antibodies or of isotype-matched control antibody (clone LTF-2, BioXCell, BE0090) on the indicated days. Specific depletion of CD4^+^ and/or CD8^+^ T cells was confirmed by FACS analysis of peripheral blood lymphocytes. To examine the effect of T cell depletion on primary tumor growth under combination therapy, mice were injected subcutaneously with 3.0 × 10^6^ KPC cells, and combination therapy was administered as described above (day 0). Tumor size was monitored, and tumors were harvested on day 14 for histological analysis. To determine the role of immune cells in secondary tumor rejection, NK cells, CD8^+^ T cells, or neutrophils were depleted by neutralizing antibodies in mice that underwent neoadjuvant therapy followed by tumor resection. Mice received 200 μg of anti-NK1.1 (clone PK136), anti-CD8 (clone 53-6.7) or anti-Ly6G (clone1A8) one day before reinoculation of KPC tumors and continuously received the antibody twice weekly^50^. Specific cell depletion was confirmed by FACS analyses of peripheral blood lymphocytes.

### Statistics

Data are presented as the mean ± s.e.m. One-way or two-way ANOVA was performed followed by Tukey’s post hoc test to compare mouse treatment groups, and the statistical significance was determined between pairs of individual groups. For all analyses, *P* < 0.05 was considered significant. All statistical analyses were carried out using GraphPad Prism version 10.2.3 (GraphPad Software). Data distribution was assumed to be normal, but this was not formally tested. No statistical methods were used to predetermine sample sizes, but our sample sizes are similar to those reported in previous publications^27,33^.

### Reporting summary

Further information on research design is available in the Nature Portfolio Reporting Summary linked to this article.

## Online content

Any methods, additional references, Nature Portfolio reporting summaries, source data, extended data, supplementary information, acknowledgements, peer review information; details of author contributions and competing interests; and statements of data and code availability are available at 10.1038/s41590-025-02259-8.

## Supplementary information

Reporting Summary

## Source data

Source Data Fig. 3 Statistical source data for Fig. 3a–d.

Source Data Fig. 8 Statistical source data for Fig. 8f.

Source Data Extended Data Fig. 4 Unprocessed western blots for Fig. 4a.

Source Data Extended Data Fig. 4 Unprocessed western blots for Fig. 4c.

## Data Availability

The single-cell and bulk RNA-seq data reported in this study are publicly available at the Gene Expression Omnibus (GEO) repository. Single-cell RNA-seq data are available at the GEO under accession numbers GSE275840 and GSE275876. The reviewer’s token for GSE275840 is afclomuwllohvah. The reviewer’s token for GSE275876 is yzqfgykuvdydfan. Mouse tumor RNA-seq data are available at the GEO under accession number GSE275836. The reviewer’s token for GSE275836 is cpyvgcwslbcbxsb. Human cancer RNA-seq data are available at the GEO under accession numbers GSE275766 for pancreatic cancer and GSE275955 for breast cancer. The reviewer’s token for GSE275766 is ebmzkqsixhshhif. The reviewer’s token for GSE275955 is cjgxuqaexnslzmr. Source data are provided with this paper.
