# Supplementary material for: Simultaneous STING and lymphotoxin-β receptor activation induces B cell responses in tertiary lymphoid structures to potentiate antitumor immunity
Source: Nat Immunol. 2025 Sep 2;26(10):1766–80. doi: 10.1038/s41590-025-02259-8 (PMC12479350; doi:10.1038/s41590-025-02259-8)
Supplement: Supplementary file 1 — Reporting Summary [file 41590_2025_2259_MOESM1_ESM.pdf]

nature portfolio

Corresponding author(s): Masanobu Komatsu

Last updated by author(s): May 26, 2025

Reporting Summary

Nature Portfolio wishes to improve the reproducibility of the work that we publish. This form provides structure for consistency and transparency in reporting. For further information on Nature Portfolio policies, see our Editorial Policies and the Editorial Policy Checklist.

Statistics

For all statistical analyses, confirm that the following items are present in the figure legend, table legend, main text, or Methods section.

n/a	Confirmed	
☐	☒	The exact sample size (*n*) for each experimental group/condition, given as a discrete number and unit of measurement
☐	☒	A statement on whether measurements were taken from distinct samples or whether the same sample was measured repeatedly
☐	☒	The statistical test(s) used AND whether they are one- or two-sided<br>*Only common tests should be described solely by name; describe more complex techniques in the Methods section.*
☒	☐	A description of all covariates tested
☐	☒	A description of any assumptions or corrections, such as tests of normality and adjustment for multiple comparisons
☐	☒	A full description of the statistical parameters including central tendency (e.g. means) or other basic estimates (e.g. regression coefficient) AND variation (e.g. standard deviation) or associated estimates of uncertainty (e.g. confidence intervals)
☐	☒	For null hypothesis testing, the test statistic (e.g. *F*, *t*, *r*) with confidence intervals, effect sizes, degrees of freedom and *P* value noted<br>*Give P values as exact values whenever suitable.*
☒	☐	For Bayesian analysis, information on the choice of priors and Markov chain Monte Carlo settings
☒	☐	For hierarchical and complex designs, identification of the appropriate level for tests and full reporting of outcomes
☒	☐	Estimates of effect sizes (e.g. Cohen's *d*, Pearson's *r*), indicating how they were calculated

Our web collection on statistics for biologists contains articles on many of the points above.

Software and code

Policy information about availability of computer code

Data collection	Flow cytometry analyses and cell sorting were done using CytoFLEX and CytoFLEX SRT (Beckman). Immunofluorescent imaging was carried out using Nikon Eclipse 90i fluorescence microscope and Nikon A1R confocal system. Stained slides were scanned by Aperio Versa (Leica) for subsequent Halo image analyses (Indica Labs). Single-cell library was generated using 10X Chromium Next GEN Single Cell 5' Kit v2, 4., followed by sequencing using Illumina NovaSeq (Illumina).
Data analysis	CytExpert software (Beckman) was used for analyzing flow cytometry data. For single-cell RNAseq, the FASTQ files were uploaded into 10X Genomics Cloud CLI (10X genomics) for alignment and mapping using Cell Ranger Count v7.1.0 with Mouse (mm10) as reference. For bulk RNAseq, Pertek®Flow® was used for trimming, QC, alignment by STAR - 2.7.8a, annotation with hg38_ensembl_release91_v2, and differential analysis with DESeq2. Quantitative image analyzes were carried out using Halo software (Indica Labs) using Cytonuclear module or Nikon NIS-Elements 5.20.01 software. Statistical analyses were performed using GraphPad Prism version 10.2.3

For manuscripts utilizing custom algorithms or software that are central to the research but not yet described in published literature, software must be made available to editors and reviewers. We strongly encourage code deposition in a community repository (e.g. GitHub). See the Nature Portfolio guidelines for submitting code & software for further information.

Data

Policy information about availability of data

All manuscripts must include a data availability statement. This statement should provide the following information, where applicable:

- Accession codes, unique identifiers, or web links for publicly available datasets
- A description of any restrictions on data availability
- For clinical datasets or third party data, please ensure that the statement adheres to our policy

Single-cell RNAseq data generated in this study are publicly available at Gene Expression Omnibus (GEO) database with accession numbers GSE275840 and GSE275876, which can be viewed at: https://www.ncbi.nlm.nih.gov/geo/query/acc.cgi?acc=GSE275840 and https://www.ncbi.nlm.nih.gov/geo/query/acc.cgi?acc=GSE275876
Bulk RNAseq data are available at GEO database with accession numbers GSE275836 for mouse tumors under different conditions, GSE275766 for pancreatic cancer and GSE275955 for breast cancer with/without TLS. These data can be viewed at:
https://www.ncbi.nlm.nih.gov/geo/query/acc.cgi?acc=GSE275836
https://www.ncbi.nlm.nih.gov/geo/query/acc.cgi?acc=GSE275766
https://www.ncbi.nlm.nih.gov/geo/query/acc.cgi?acc=GSE275955

Research involving human participants, their data, or biological material

Policy information about studies with human participants or human data. See also policy information about sex, gender (identity/presentation), and sexual orientation and race, ethnicity and racism.

Reporting on sex and gender	Previously archived breast cancer and pancreatic cancer tissue FFPE sections were used in this study. Breast cancer specimens were from female patients, and pancreatic cancer specimens were from both male and female patients.
Reporting on race, ethnicity, or other socially relevant groupings	Neither race nor ethnicity was considered as a study criterion.
Population characteristics	Previously archived cancer tissue FFPE sections were from cancer patients of age 32-71.
Recruitment	Existing FFPE specimens archived at the National Cancer Center Hospital, Japan were used for the study.
Ethics oversight	Institutional Review Board (IRB protocol# 2005-077) National Cancer Center Hospital, Japan

Note that full information on the approval of the study protocol must also be provided in the manuscript.

Field-specific reporting

Please select the one below that is the best fit for your research. If you are not sure, read the appropriate sections before making your selection.

☒ Life sciences ☐ Behavioural & social sciences ☐ Ecological, evolutionary & environmental sciences

For a reference copy of the document with all sections, see nature.com/documents/nr-reporting-summary-flat.pdf

Life sciences study design

All studies must disclose on these points even when the disclosure is negative.

Sample size	The number of mice used for each experiment was chosen based on our pilot experiments and previously published work by others conducting similar analyses. The chosen sample sizes were sufficient to determine statistical significance of P < 0.05.
Data exclusions	For single-cell RNAseq analyses, cells expressing <300 genes or >5,000 genes and cells whose mitochondrial content > 6% were excluded. The data points of CD45-negative cells were excluded in the analysis of CD45+ sorted cells. For the analysis of human pancreatic cancer, adenocarcinomas originating in intraductal papillary mucinous neoplasms or mucinous cystic neoplasms were excluded, as were secondary tumors and post-neoadjuvant cases. Autoimmune pancreatitis associated cancers were excluded.
Replication	Experiments were repeated once or more times unless indicated otherwise in figure legends. All replications were successful.
Randomization	Tumor-bearing were randomized to have the initial average tumor burden to be approximately equal between groups.
Blinding	Investigators were not blinded for logistical reasons: the same investigator was responsible for carrying out the study, setting up experiments, acquiring, and analyzing the data. However, a different investigator was involved in ensuring the accuracy of the results.

Reporting for specific materials, systems and methods

We require information from authors about some types of materials, experimental systems and methods used in many studies. Here, indicate whether each material, system or method listed is relevant to your study. If you are not sure if a list item applies to your research, read the appropriate section before selecting a response.

Materials & experimental systems

n/a	Involved in the study
☐	☒ Antibodies
☐	☒ Eukaryotic cell lines
☒	☐ Palaeontology and archaeology
☐	☒ Animals and other organisms
☐	☒ Clinical data
☒	☐ Dual use research of concern
☒	☐ Plants

Methods

n/a	Involved in the study
☐	☐ ChIP-seq
☐	☒ Flow cytometry
☐	☐ MRI-based neuroimaging

Antibodies

Antibodies used

ANTIBODIES / SOURCE / IDENTIFIER
APC anti-mouse CD19 Recombinant Antibody Biolegend 159806
Brilliant Violet 785 anti-mouse/human CD44 Biolegend 103059
Alexa Fluor 488 anti-mouse CD69 Biolegend 104516
PE anti-mouse CD138 (Syndecan-1) Antibody BioLegend 142504
FITC anti-mouse CD73 Antibody Biolegend 127220
Brilliant Violet 421 anti-mouse CD273(PDL2 ,B7-DC) Biolegend 107219
Alexa Fluor 647 anti-mouse CD3 Biolegend 100209
Brilliant Violet 421 anti-mouse CD4 Biolegend 100438
Alexa Fluor 488 anti-mouse CD8a Biolegend 100723
APC anti-mouse CD62L Antibody Biolegend 104412
Ghost Dye Violet 510 Viability Dye CYTEK SKU 13-0870-T100
anti-mouse IgM-Alexa488 (goat) Thermo Fisher Scientific A21042
anti-mouse IgG-Alexa 647 (goat) Thermo Fisher Scientific A21236
anti-rabbit IgG-Alexa 555 Thermo Fisher Scientific
anti-mouse IgG Alexa 555 Thermo Fisher Scientific A32727
anti-rat IgG Alexa 488 Thermo Fisher Scientific A11006
Purified anti-mouse/human PNAd Antibody, MECA-79 Bioledgends 120802
anti-CD19 Antibody, Rabbit Monoclonal SB SinoBiological 50510-R014
anti-EPCAM Abcam ab71916
antio-mouse CD8 (Clone 53-6.7) Bioledgends 100724
anti-mouse CD23 antibody Thermo Fisher PA5-79242
anti-CD21 antibody Novus Biological NBP2-67605
Anti-EpCAM antibody (ab71916) Abcam ab71916
anti-mouse CD138 (Syndecan-1) Antibody Bioledgends 142502
anti-mouse CD4 antibody-Alexa488 Biolegend 100423
anti-Bcl6 antibody Abcam ab272859
anti-Ki67 antibody (Rabbit, clone D3B5) Cell Singaling 12202S
anti-CD3 antibody (SP7) rabbit monoclonal Thermo Fisher Scientific MA1-90582
anti-mouse CD38 (Syndecan-1) antibody Bioledgends 142502
anti -mosue CD19-AF647 BD Bioscience 557684
Alexa Fluor® 700 anti-mouse CD45 Antibody Biolegend 103127
FITC anti-mouse CD3ε Antibody Biolegend 100306
Brilliant Violet 421™ anti-mouse CD4 Antibody Biolegend 100437
Brilliant Violet 510™ anti-mouse CD8a Antibody Biolegend 100752
PerCP/Cyanine5.5 anti-mouse/human CD11b Antibody Biolegend 101228
Brilliant Violet 785™ anti-mouse Ly-6G Antibody Biolegend 127645

Validation

All antibodies used in this study were from commercial sources, and validation information is available at the product information page of each company's website, which can be found with the catalog No. indicated above.

Eukaryotic cell lines

Policy information about cell lines and Sex and Gender in Research

Cell line source(s)

Py230 mammary tumor cell line was purchased from ATCC (#CRL-3279); the KPC mouse-derived KxPxCx pancreatic cell line was a gift from Dr. Elizabeth M. Jaffee, Johns Hopkins University; 76-9 rhabdomyosarcoma line was obtained from National Cancer Institute

Authentication

The sex of each cell line was confirmed by PCR. Cell lines were not authenticated in house after procured from the original source. However, each cell line was maintained separately and stored in early passages to preserve cell purity.

Mycoplasma contamination

The Py230 line was purchased free of mycoplasma contamination as certified by ATCC.

Mycoplasma contamination	The other two lines were not tested in house for mycoplasma contamination since obtaining from the original source.
Commonly misidentified lines (See ICLAC register)	None

Animals and other research organisms

Policy information about studies involving animals; ARRIVE guidelines recommended for reporting animal research, and Sex and Gender in Research

Laboratory animals	C57BL/6NHsd mice at 8-12 weeks old were purchased from ENVIGO (Order Code: 044). Nude mice with C57BL/6 background (B6.Cg-Foxn1nu/J) were obtained from the Jackson Laboratory (Strain #:000819).
Wild animals	None
Reporting on sex	To match sex of tumor cells with the recipient mice, male mice were used for KxPxCx implantation, and female mice were used for Py230 or 76-9 implantation.
Field-collected samples	N/A
Ethics oversight	Institutional Animal Care and Use Committee of Johns Hopkins University (Protocol No. MO21C338)

Note that full information on the approval of the study protocol must also be provided in the manuscript.

Clinical data

Policy information about clinical studies

All manuscripts should comply with the ICMJE guidelines for publication of clinical research and a completed CONSORT checklist must be included with all submissions.

Clinical trial registration	*Provide the trial registration number from ClinicalTrials.gov or an equivalent agency.*
Study protocol	*Note where the full trial protocol can be accessed OR if not available, explain why.*
Data collection	*Describe the settings and locales of data collection, noting the time periods of recruitment and data collection.*
Outcomes	*Describe how you pre-defined primary and secondary outcome measures and how you assessed these measures.*

Plants

Seed stocks	*Report on the source of all seed stocks or other plant material used. If applicable, state the seed stock centre and catalogue number. If plant specimens were collected from the field, describe the collection location, date and sampling procedures.*
Novel plant genotypes	*Describe the methods by which all novel plant genotypes were produced. This includes those generated by transgenic approaches, gene editing, chemical/radiation-based mutagenesis and hybridization. For transgenic lines, describe the transformation method, the number of independent lines analyzed and the generation upon which experiments were performed. For gene-edited lines, describe the editor used, the endogenous sequence targeted for editing, the targeting guide RNA sequence (if applicable) and how the editor was applied.*
Authentication	*Describe any authentication procedures for each seed stock used or novel genotype generated. Describe any experiments used to assess the effect of a mutation and, where applicable, how potential secondary effects (e.g. second site T-DNA insertions, mosiacism, off-target gene editing) were examined.*

ChIP-seq

Data deposition

☐ Confirm that both raw and final processed data have been deposited in a public database such as GEO.

☐ Confirm that you have deposited or provided access to graph files (e.g. BED files) for the called peaks.

Data access links *May remain private before publication.*	*For "Initial submission" or "Revised version" documents, provide reviewer access links. For your "Final submission" document, provide a link to the deposited data.*
Files in database submission	*Provide a list of all files available in the database submission.*
Genome browser session (e.g. UCSC)	*Provide a link to an anonymized genome browser session for "Initial submission" and "Revised version" documents only, to enable peer review. Write "no longer applicable" for "Final submission" documents.*

Methodology

Replicates	*Describe the experimental replicates, specifying number, type and replicate agreement.*
Sequencing depth	*Describe the sequencing depth for each experiment, providing the total number of reads, uniquely mapped reads, length of reads and whether they were paired- or single-end.*
Antibodies	*Describe the antibodies used for the ChIP-seq experiments; as applicable, provide supplier name, catalog number, clone name, and lot number.*
Peak calling parameters	*Specify the command line program and parameters used for read mapping and peak calling, including the ChIP, control and index files used.*
Data quality	*Describe the methods used to ensure data quality in full detail, including how many peaks are at FDR 5% and above 5-fold enrichment.*
Software	*Describe the software used to collect and analyze the ChIP-seq data. For custom code that has been deposited into a community repository, provide accession details.*

Flow Cytometry

Plots

Confirm that:

☒ The axis labels state the marker and fluorochrome used (e.g. CD4-FITC).

☒ The axis scales are clearly visible. Include numbers along axes only for bottom left plot of group (a 'group' is an analysis of identical markers).

☒ All plots are contour plots with outliers or pseudocolor plots.

☒ A numerical value for number of cells or percentage (with statistics) is provided.

Methodology

Sample preparation	For the analyses of tumor-infiltrating lymphocytes, tumors were excised and cut into smaller fragments. These fragments underwent enzymatic digestion with Liberase™ (300 µg/mL) and mechanical dissociation using the GentleMACS Octo dissociator (protocol: hard tumor dissociation, TDK-3, 1 hour at 37°C). Following dissociation, the cell suspension was filtered using a 70 µm strainer and then washed twice with RPMI medium at 400 rcf for 5 minutes. Live leukocytes were then stained with cell marker antibodies.
Instrument	Beckman Coulter CytoFLEX LX Flow Cytometer and CytoFLEX SRT Cell Sorter
Software	Beckman Coulter CytExpert Analysis Software Version 2.5.0.77
Cell population abundance	For 10X Chromium, 10 tumors were pooled for each treatment group, and a total of 1x107 cells were sorted to yield CD45+ cell population with 97-98% post-sort purity. 20,000 sorted cells were used for single-cell analysis.
Gating strategy	Gating strategies are shown in supplemental data figures

☒ Tick this box to confirm that a figure exemplifying the gating strategy is provided in the Supplementary Information.

Magnetic resonance imaging

Experimental design

Design type	*Indicate task or resting state; event-related or block design.*
Design specifications	*Specify the number of blocks, trials or experimental units per session and/or subject, and specify the length of each trial or block (if trials are blocked) and interval between trials.*
Behavioral performance measures	*State number and/or type of variables recorded (e.g. correct button press, response time) and what statistics were used to establish that the subjects were performing the task as expected (e.g. mean, range, and/or standard deviation across subjects).*

Acquisition

Imaging type(s) | *Specify: functional, structural, diffusion, perfusion.*

Field strength | *Specify in Tesla*

Sequence & imaging parameters | *Specify the pulse sequence type (gradient echo, spin echo, etc.), imaging type (EPI, spiral, etc.), field of view, matrix size, slice thickness, orientation and TE/TR/flip angle.*

Area of acquisition | *State whether a whole brain scan was used OR define the area of acquisition, describing how the region was determined.*

Diffusion MRI ☐ Used ☐ Not used

Preprocessing

Preprocessing software | *Provide detail on software version and revision number and on specific parameters (model/functions, brain extraction, segmentation, smoothing kernel size, etc.).*

Normalization | *If data were normalized/standardized, describe the approach(es): specify linear or non-linear and define image types used for transformation OR indicate that data were not normalized and explain rationale for lack of normalization.*

Normalization template | *Describe the template used for normalization/transformation, specifying subject space or group standardized space (e.g. original Talairach, MNI305, ICBM152) OR indicate that the data were not normalized.*

Noise and artifact removal | *Describe your procedure(s) for artifact and structured noise removal, specifying motion parameters, tissue signals and physiological signals (heart rate, respiration).*

Volume censoring | *Define your software and/or method and criteria for volume censoring, and state the extent of such censoring.*

Statistical modeling & inference

Model type and settings | *Specify type (mass univariate, multivariate, RSA, predictive, etc.) and describe essential details of the model at the first and second levels (e.g. fixed, random or mixed effects; drift or auto-correlation).*

Effect(s) tested | *Define precise effect in terms of the task or stimulus conditions instead of psychological concepts and indicate whether ANOVA or factorial designs were used.*

Specify type of analysis: ☐ Whole brain ☐ ROI-based ☐ Both

Statistic type for inference | *Specify voxel-wise or cluster-wise and report all relevant parameters for cluster-wise methods.*

(See Eklund et al. 2016)

Correction | *Describe the type of correction and how it is obtained for multiple comparisons (e.g. FWE, FDR, permutation or Monte Carlo).*

Models & analysis

n/a	Involved in the study
☐	☐ Functional and/or effective connectivity
☐	☐ Graph analysis
☐	☐ Multivariate modeling or predictive analysis

Functional and/or effective connectivity | *Report the measures of dependence used and the model details (e.g. Pearson correlation, partial correlation, mutual information).*

Graph analysis | *Report the dependent variable and connectivity measure, specifying weighted graph or binarized graph, subject- or group-level, and the global and/or node summaries used (e.g. clustering coefficient, efficiency, etc.).*

Multivariate modeling and predictive analysis | *Specify independent variables, features extraction and dimension reduction, model, training and evaluation metrics.*
