# Supplementary figures and images for: Simultaneous STING and lymphotoxin-β receptor activation induces B cell responses in tertiary lymphoid structures to potentiate antitumor immunity
Source: Nat Immunol. 2025 Sep 2;26(10):1766–80. doi: 10.1038/s41590-025-02259-8 (PMC12479350; doi:10.1038/s41590-025-02259-8)

KPC tumor

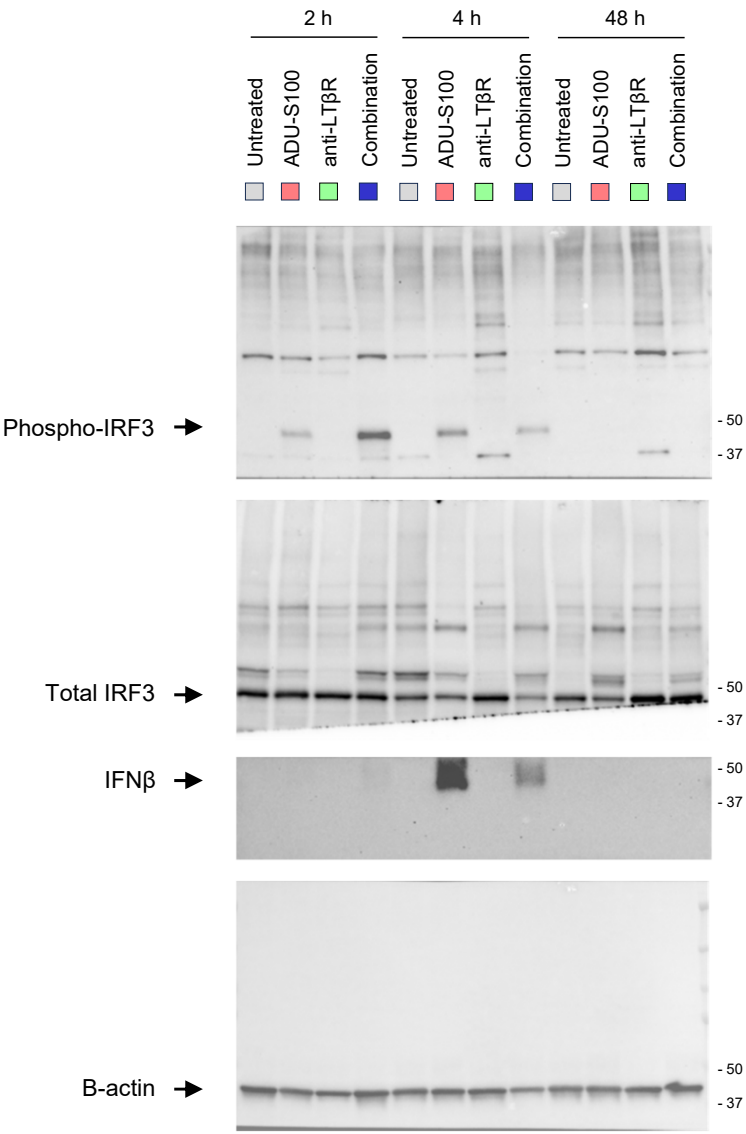

Supplement: Supplementary file 4 — Unprocessed western blots for Fig. 4a. [file 41590_2025_2259_MOESM4_ESM.pdf]

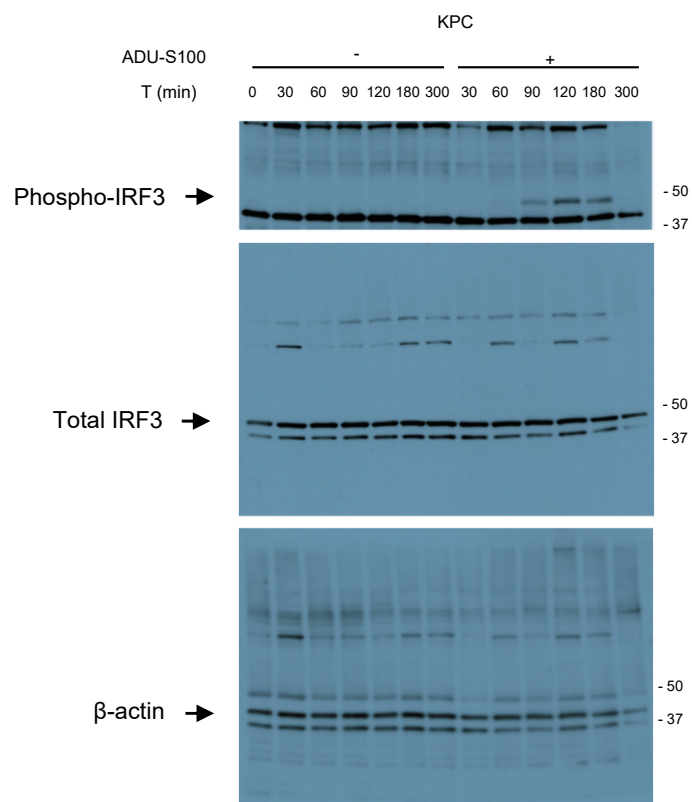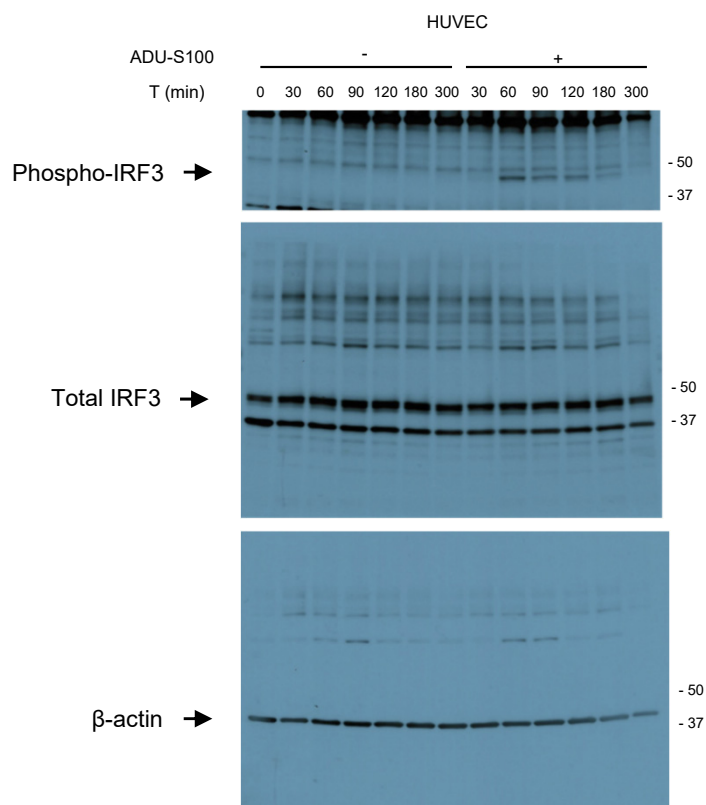

Supplement: Supplementary file 5 — Unprocessed western blots for Fig. 4c. [file 41590_2025_2259_MOESM5_ESM.pdf]
